# Proteasome‐dependent degradation of histone H1 subtypes is mediated by its C‐terminal domain

**DOI:** 10.1002/pro.4970

**Published:** 2024-04-09

**Authors:** D. García‐Gomis, J. López, A. Calderón, M. Andrés, I. Ponte, A. Roque

**Affiliations:** ^1^ Biochemistry and Molecular Biology Department, Biosciences Faculty Universitat Autònoma de Barcelona Barcelona Spain

**Keywords:** 20S proteasome, histone H1, intrinsically disordered domains, PA28, phosphorylation, proteasome inhibitors

## Abstract

Histone H1 is involved in chromatin compaction and dynamics. In human cells, the H1 complement is formed by different amounts of somatic H1 subtypes, H1.0‐H1.5 and H1X. The amount of each variant depends on the cell type, the cell cycle phase, and the time of development and can be altered in disease. However, the mechanisms regulating H1 protein levels have not been described. We have analyzed the contribution of the proteasome to the degradation of H1 subtypes in human cells using two different inhibitors: MG132 and bortezomib. H1 subtypes accumulate upon treatment with both drugs, indicating that the proteasome is involved in the regulation of H1 protein levels. Proteasome inhibition caused a global increase in cytoplasmatic H1, with slight changes in the composition of H1 bound to chromatin and chromatin accessibility and no alterations in the nucleosome repeat length. The analysis of the proteasome degradation pathway showed that H1 degradation is ubiquitin‐independent. The whole protein and its C‐terminal domain can be degraded directly by the 20S proteasome *in vitro*. Partial depletion of PA28γ revealed that this regulatory subunit contributes to H1 degradation within the cell. Our study shows that histone H1 protein levels are under tight regulation to prevent its accumulation in the nucleus. We revealed a new regulatory mechanism for histone H1 degradation, where the C‐terminal disordered domain is responsible for its targeting and degradation by the 20S proteasome, a process enhanced by the regulatory subunit PA28γ.

## 1 INTRODUCTION

Histone H1 is a multigene family associated with the regulation of chromatin structure. In humans, the H1 family is composed of 11 subtypes or variants. Seven subtypes (H1.0‐H1.5, H1X) are differentially expressed in somatic cells, while the remaining four are germ‐line specific (Talbert et al., 2012). Somatic subtypes are subdivided into two groups: replication‐dependent (RD) and replication‐independent (RI), according to their expression patterns during cell cycle (Duronio & Marzluff, 2017; Millán‐Ariño et al., 2016).

Histone H1 subtypes are basic proteins with three structural domains: the N‐terminal domain (NTD), the globular domain (GD), and the C‐terminal domain (CTD). The GD has approximately 80 residues and a stably folded (Cerf et al., 1993; Ramakrishnan et al., 1993). It is responsible for H1 binding to the nucleosome dyad and it is highly conserved in evolution (Ponte et al., 1998; Ramakrishnan et al., 1993). The NTD is a short domain of 20–36 residues, while the CTD is the longest domain with about 100 residues. Both terminal domains are intrinsically disordered enriched in proline, serine, alanine, and especially lysine (Hansen et al., 2006; Lu & Hansen, 2004; Roque et al., 2005; Vila et al., 2001, 2002). The CTD is the main determinant of chromatin compaction within histone H1 (Hendzel et al., 2004). This domain contains several cyclin‐dependent kinases (CDK) consensus sites, which are modified in a cell cycle‐dependent manner and affect the secondary structure of the CTD bound to DNA and chromatin, as well as its interaction with chromatin (Lopez et al., 2015; Raghuram et al., 2013; Roque et al., 2008). H1 phosphorylation also facilitates nuclear export (Bleher & Martin, 1999).

The protein levels of histone H1 are tightly regulated during development as they can alter chromatin compaction and transcription. In stem cells, there is approximately one H1 molecule in every two nucleosomes (H1: nucleosome ratio of 0.5), favoring chromatin accessibility and high transcriptional activity (Fan et al., 2005; Zhang et al., 2012). In adults, the H1: nucleosome ratio increases, with values between 0.8 and 1 (Pan & Fan, 2016). In cells with very low transcriptional activity, like chicken erythrocytes, this ratio increases up to 1.3, promoting gene silencing (Bates & Thomas, 1981; Beacon & Davie, 2021; Delcuve & Davie, 1989). Gene knockout or knockdown affecting one or two H1 subtypes is not lethal, as the loss is compensated by other subtypes, usually H1.0 (Fan et al., 2003; Izquierdo‐Bouldstridge et al., 2017). However, triple knockout of H1.2, H1.3, and H1.4 in mice is deleterious during gestation, and the embryonic stem cells derived from these embryos had alterations in the nucleosome spacing and impaired differentiation (Fan et al., 2003; Zhang et al., 2012). Therefore, it is interesting to study the regulatory mechanisms controlling H1 protein levels.

Proteolysis is a crucial regulatory mechanism in the maintenance of proteostasis. Three mechanisms contribute to protein degradation: proteases, the lysosomal system, and the proteasome complex. Proteasomal degradation is responsible for the elimination of most damaged, misfolded, or unfolded proteins in the cell in the nucleus and the cytoplasm (Peters et al., 1994). The proteolytic activity is within a barrel‐shaped protein complex known as the 20S proteasome or core particle, which can associate with different regulatory particles on one or both ends (Fricker, 2020).

There are two pathways of proteasomal degradation of proteins: ubiquitin (Ub)‐dependent and Ub‐independent. Ub‐dependent proteolysis is mediated by the 26S proteasome, formed by the 20S proteasome and the 19S regulatory particle, and the targeting of substrates involves the addition of one or more ubiquitin monomers (Shabek et al., 2012). Proteins degraded by the Ub‐independent proteasomal pathway are recognized by different signals, such as specific amino acid sequences (Murakami et al., 1992), post‐translational modifications (Qian et al., 2013), or disordered regions enriched in basic and flexible amino acids (Kudriaeva et al., 2019). Degradation is carried out directly by the 20S proteasome or coupled with PA28 or PA200 regulatory particles (Cascio, 2021; Jiang et al., 2021).

Core histones are long‐lived proteins, but their degradation is triggered under specific conditions, such as response to EGF, DNA damage, and oxidative stress (reviewed in (Dhaenens et al., 2015; Shmueli et al., 2022). In contrast, the proteolytic mechanisms involved in the regulation of histone H1 protein levels are largely unexplored. Early studies showed that the turnover rate of histone H1 is higher than that of core histones, and its magnitude is variable depending on the replicative state of the cells. H1 half‐live in rat brain changed from 13 to 112 days between neonate and adult animals, while its value decreased to hours in K562 cells in culture (Duerre & Lee, 1974; Ullrich & Grune, 2001). The degradation rate of histone H1 was enhanced after exposure to oxidative conditions by the action of the 20S proteasome. In these conditions, activation of PARP1 accelerated H1 degradation (Ullrich & Grune, 2001). Induction of glycoxidation promoted H1 degradation by the nuclear proteasome (Cervantes‐Laurean et al., 2005). Histone H1 proteolysis is also enhanced by gamma‐irradiation and TNF‐induced apoptosis (McConkey, 1996; Voelkel‐Johnson et al., 1995).

In the present work, we have studied the contribution of the proteasome to the regulation of histone H1 protein levels. Using proteasome inhibitors, we have analyzed the accumulation of H1 subtypes, the changes in subcellular distribution, and the effect on chromatin. We have also examined the pathway of proteasomal degradation and the contribution of histone H1 structural domains.

## 2 RESULTS

### 2.1 Effect of proteasome inhibitors in the protein levels of H1 subtypes

We analyzed the role of the proteasome in the control of the protein levels of histone H1 subtypes by inhibition experiments. The proteasome inhibition was assessed using the peptide Suc‐LLVY‐AMC, which releases the fluorophore after digestion by the chymotrypsin‐like activity of the proteasome (Zafar et al., 2007). The results showed that treatment with MG132 inhibited proteasome activity at the selected dose (Figure S1).

**FIGURE 1:**
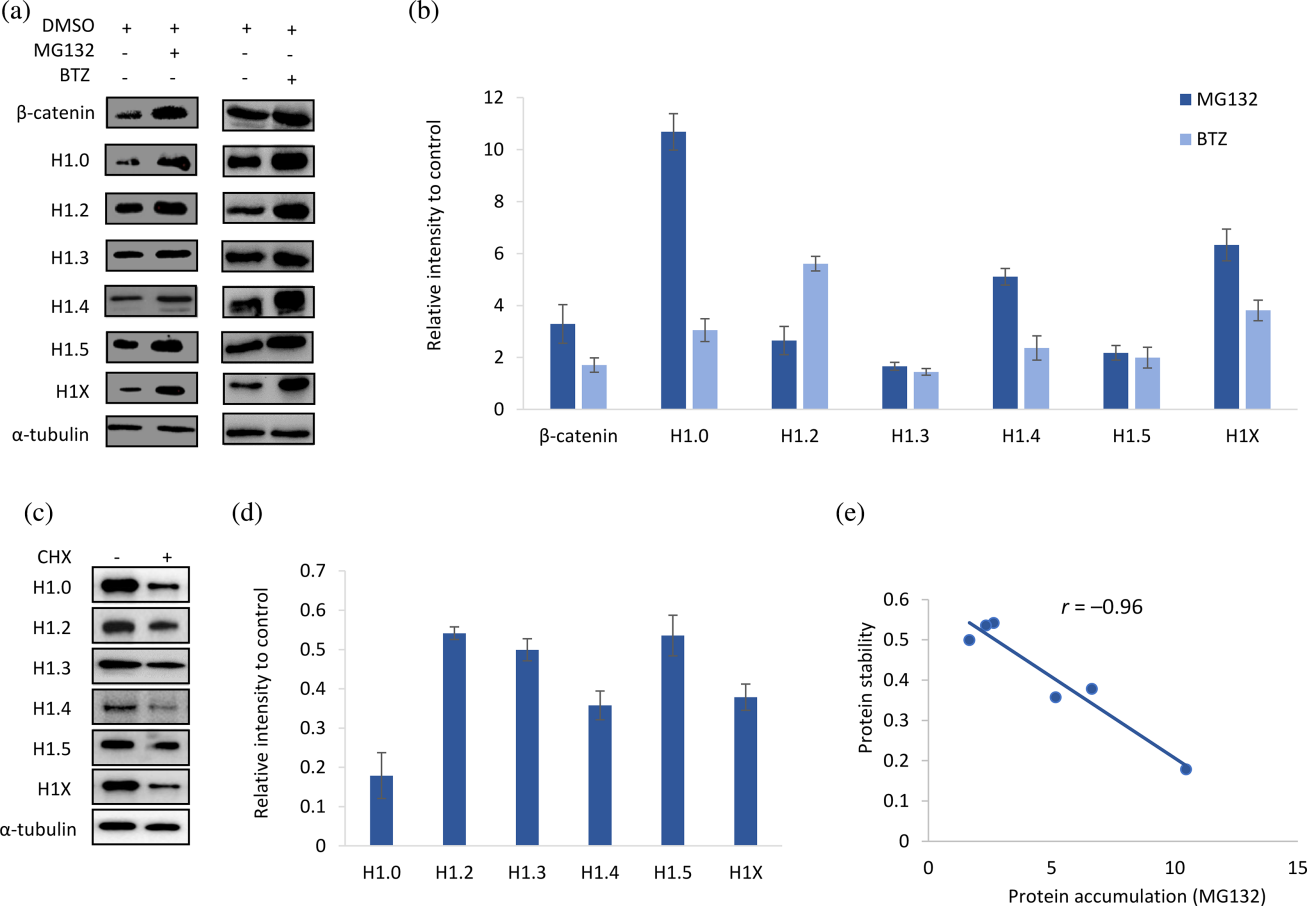
Effects of proteasome inhibition on Histone H1 variants in T47D cells. (a) Western Blots of total protein extracts of T47D cells treated with DMSO, MG132 (20 μM 12 h), and Bortezomib (20 nM 12 h). (c) Western Blots of total protein extract after translation inhibition treatment by cycloheximide (10 μg/mL 8 h). (b, d) Quantification of the Western blot images of three biological replicates corresponding to (a) and (c), respectively. Error bars correspond to the standard deviation. (e) Scatter plot and correlation between protein accumulation and protein stability.

The effect on the protein levels was analyzed by Western blot. Treatment with MG132 caused an accumulation of β‐catenin, used as a positive control (Aberle et al., 1997). We observed an accumulation of all the expressed H1 subtypes in T47D and HeLa (Figure 1a; Figure S2). Subtype H1.3 is not detectable in HeLa, while H1.1 cannot be detected in both cell lines. We found a differential accumulation of somatic H1 subtypes with the highest increase in H1.0, followed by H1X (Figure 1b; Figure S2).

MG132 inhibits the proteolytic activity of the proteasome and calpains, so we also used bortezomib (BTZ), a specific proteasome inhibitor (Fricker, 2020; Goldberg, 2012). In the presence of bortezomib, all H1 subtypes accumulated, confirming the role of the proteasome in regulating H1 protein levels (Figure 1a). The level accumulation of H1 subtypes was slightly lower than in MG132, except for H1.2. These results suggested that other proteolytic mechanisms might contribute to the degradation of H1 subtypes.

The distinct accumulation of H1 subtypes upon proteasome inhibition may be explained by differences in protein stability. In T47D, inhibition of protein translation with cycloheximide caused a decrease in the protein levels of H1 subtypes, albeit in different proportions (Figure 1c,d). The highest decrease was observed in H1.0, the subtype with the highest accumulation in MG132. Protein accumulation of H1 subtypes upon proteasome inhibition had a negative correlation (*r* = −0.96) with the protein fraction remaining after translation inhibition (Figure 1e). Furthermore, the combined treatment with cycloheximide and MG132 resulted in the maintenance of H1 protein levels (Figure S3). In HeLa, we observed a similar correlation between the accumulation of H1 subtypes and their stability (*r* = −0.99) (Figure S4). In this cell line, H1.0 was also the subtype with the highest accumulation, but its stability could not be measured due to the low levels present at the initial conditions. Our results suggest that H1 subtypes with a higher protein accumulation are less stable, so their protein levels depend on their translation rate.

Protein accumulation may be modulated at the transcript level, so we analyzed the effect of proteasome inhibition in the mRNA levels of H1 subtypes. Treatment with MG132 caused a decrease in the transcript levels of all H1 subtypes in HeLa and T47D (Figure S5). We observed a reduction of more than 40% for the transcripts of H1.0 and H1X and of more than 70% for the replication‐dependent subtypes. Considering that H1 transcription is coupled to cell cycle we analyzed if it was affected by treatment with MG132. We found no significant changes in the proportions of cell cycle phases in T47D upon proteasome inhibition and a slight decrease in cell survival that could not fully account for the changes in the mRNA levels (Figure S6). Two main causes could explain the changes described above: (1) a feedback regulatory loop triggered by the increase in the protein levels, and (2) indirect effects of the drug.

### 2.2 Distribution of H1 subtypes after proteasome inhibition

**FIGURE 2:**
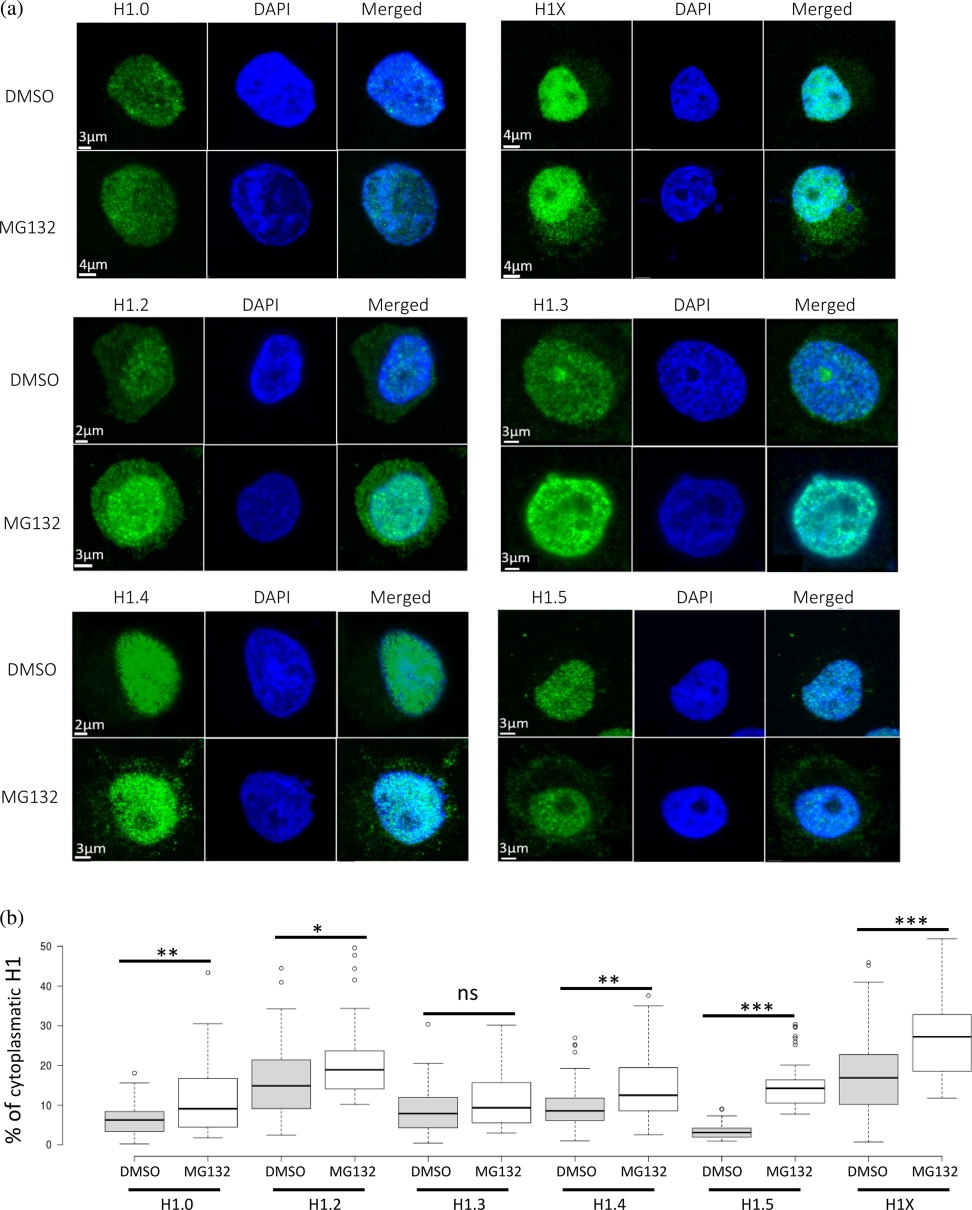
Accumulation of histone H1 subtypes in the cytoplasm of T47D cells after proteasome inhibition with MG132. (a) Representative immunofluorescence images of histone H1 somatic subtypes on T47D cells. Cells were treated with DMSO and MG132 (20 μM 12 h). The cell nucleus was stained with DAPI. (b) Box plots correspond to the quantification of 35–70 cells/subtype and condition. Asterisks denote the p‐value of the two‐tailed Student's *t*‐test showing the significance of the difference between untreated and treated cells *p‐value <0.05; **p‐value <0.01; ***p‐value <0.001; n.s, not significant.

Histone H1 is mainly a nuclear protein, but its presence in the cytoplasm could be detected by immunofluorescence (Zlatanova et al., 1990). The levels detected in the cytoplasm of T47D cells differed depending on the subtype. The estimated percentages of cytoplasmatic H1.0 and H1.5 were less than 10%, approximately 10% for H1.3 and H1.4, and more than 15% for H1.2 and H1X (Figure 2). After treatment with MG132, the amount of all H1 subtypes increased in the cytoplasm, in particular for H1.5 (Figure 2). The increase was statistically significant, except for H1.3. This change in the distribution of H1 subtypes to the cytoplasmatic fraction was confirmed by Western blot (Figure S7). Histone H3 was also detected in the cytoplasm, but at a similar level in both conditions, indicating that the increase in H1 was not due to altered permeabilization of the nuclear membrane or the disruption of chromatin structure (Figure S7). The accumulation in the cytoplasm was observed for all subtypes in T47D cells treated with BTZ (Figure S8) and for H1.2 in HeLa treated with MG132 (Figure S9). The increase was statistically significant, except for H1.0 and H1X in BTZ (Figures S8 and S9).

**FIGURE 3:**
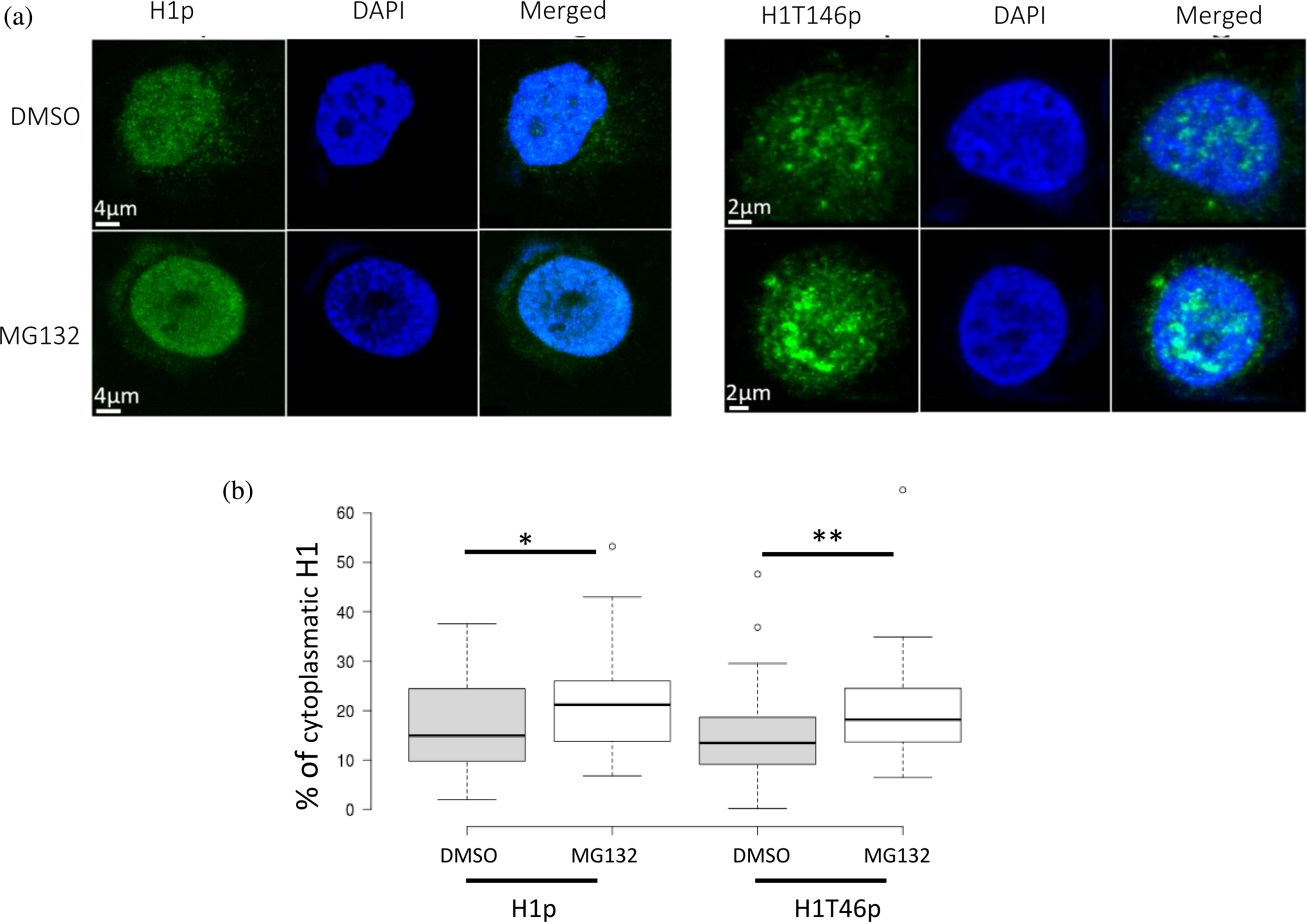
Accumulation of phosphorylated H1 in the cytoplasm of T47D cells after proteasome inhibition with MG132. (a) Representative immunofluorescence images of H1p and H1T56p on T47D cells. Cells were treated with DMSO and MG132 (20 μM 12 h). The cell nucleus was stained with DAPI. (b) Box plots correspond to the quantification of 35–70 cells/modification and condition. Asterisks denote the *p*‐value of the two‐tailed Student's *t*‐test showing the significance of the difference between untreated and treated cells *p‐value <0.05; **p‐value <0.01.

The presence of post‐translational modifications (PTMs), in particular phosphorylation, can alter H1 affinity for chromatin, as well as its subcellular localization (Bleher & Martin, 1999; Bolton & Betmouni, 1999). We used two antibodies against phosphorylated H1 to analyze if there were changes in its subcellular distribution upon proteasome inhibition (Figure 3a). The first antibody recognizes hyperphosphorylated H1, while the second antibody recognizes H1.4T146p. The percentages of phosphorylated H1 in the cytoplasm were higher than 15% and comparable in magnitude to the more abundant subtypes in the cytoplasm. The addition of MG132 caused a significant increase in the cytoplasmic levels of phosphorylated H1 (Figure 3b). The relative increase of both types of phosphorylation suggests that this modification could contribute to the accumulation of H1 in the cytoplasm, although it may not be the only cause.

### 2.3 Effects of proteasome inhibition in chromatin

**FIGURE 4:**
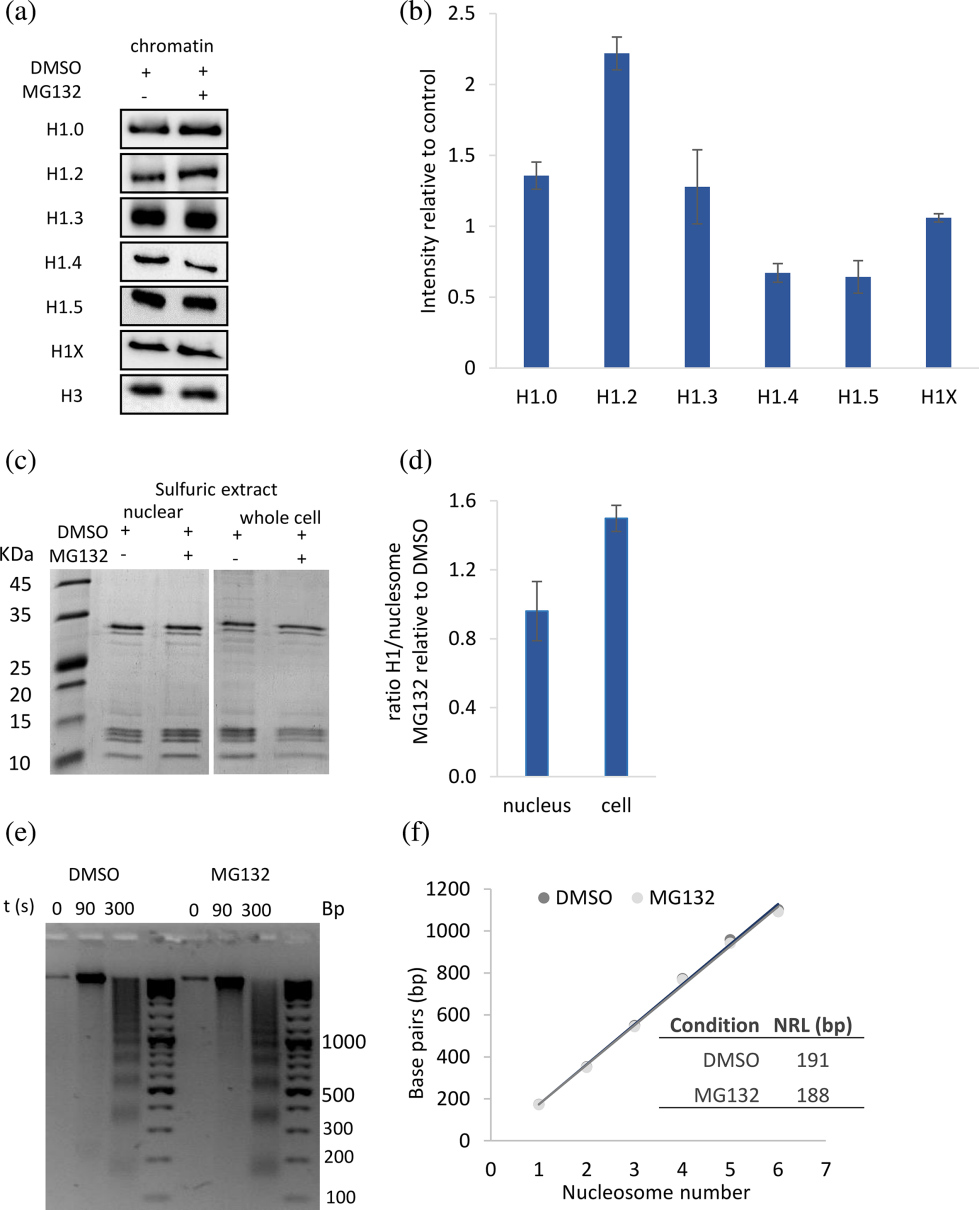
Effects at chromatin level of the changes in histone H1 subtypes upon proteasome inhibition. (a) Western Blots and quantification of chromatin‐bound H1. (b) Quantification of the Western blot images. (c) SDS‐PAGE of total histone extractions from whole cells and isolated nuclei. (d) Fold change of the H1: nucleosome ratio between MG132 and DMSO in whole cells and nuclear extracts. (e) Digestion with micrococcal nuclease of chromatin of cells treated with DMSO and MG132. (f) Nucleosome repeat length calculated from the digestions shown in (e). In all the experiments cells were treated with DMSO as a negative control or with MG132 (20 μM 12 h). Error bars correspond to standard deviation of three biological replicates.

The accumulation of H1 subtypes may alter chromatin structure and compaction. We analyzed the changes in the H1 bound to chromatin after proteasome inhibition using Western blot (Figure 4a,b). Treatment with MG132 caused a rearrangement in the proportion of the subtypes bound to chromatin. The amount of H1.0 and H1.2 bound to chromatin increased, while that of H1.4 and H1.5 decreased. Subtypes H1.3 and H1X remained unaltered.

To assess global changes in the nuclear H1 content we determined the H1: nucleosome ratio using sulfuric acid extractions of the isolated nuclei and whole cells. We found that the ratio H1: nucleosome increased 1.5‐fold in the whole cell extract, while in the nucleus it was 0.96‐fold, almost identical to the untreated (Figure 4c,d). These findings confirmed the increase in the cytoplasm observed by immunofluorescence. We analyzed if there were changes in chromatin accessibility after proteasome inhibition with MG132 by digestion with micrococcal nuclease. The digestion pattern was similar between the two samples, although a slight increase in accessibility could be observed (Figure 4e). However, the nucleosome repeat length (NRL) remained unaltered (Figure 4f). These results suggest that the accumulation of H1 in the cytoplasm after proteasome inhibition prevented significant changes in chromatin structure and compaction.

### 2.4 Mechanism of degradation of histone H1 subtypes by the proteasome

Protein degradation by the proteasome can occur through different pathways. The most common one is the degradation of ubiquitinated proteins by 26S proteasome. Protein mono‐ or polyubiquitination causes an increase in the molecular weight of the targeted proteins. In the case of H1, we detected an accumulation of all subtypes after treatment with proteasome inhibitors. However, the electrophoretic mobility of H1s remained unaltered, and no bands of higher molecular weight were observed, suggesting that the contribution of the ubiquitin‐dependent proteasome degradation may not be its main degradation pathway.

**FIGURE 5:**
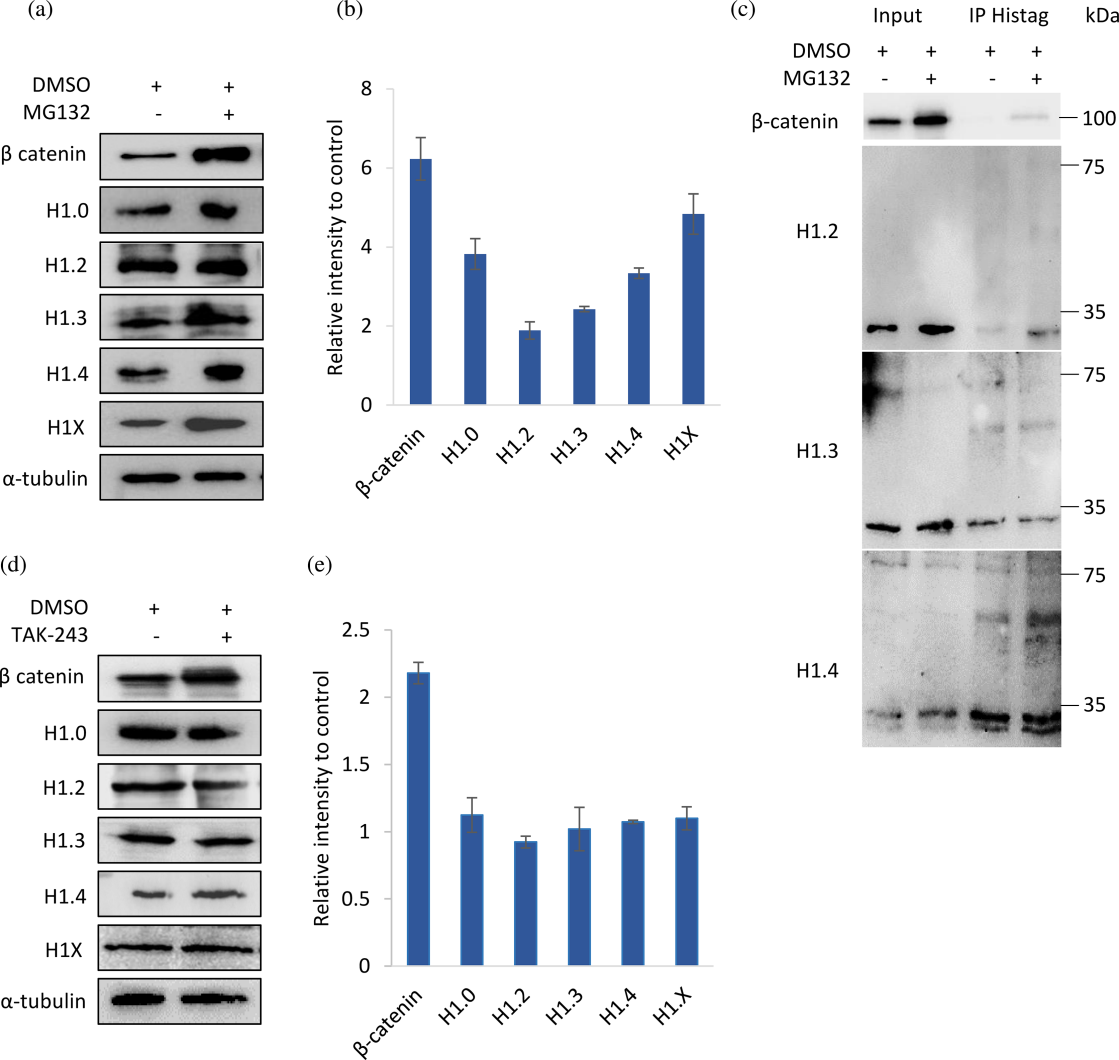
Contribution of the ubiquitin‐dependent pathway to H1 degradation. (a, b) Western Blot and quantification of total protein extracts upon proteasome inhibition with MG132 (20 μM 12 h). (c) Western blot of anti‐His immunoprecipitated proteins from HEK293T cells carrying a His‐tagged ubiquitin and treated with DMSO or MG132. (d, e) Western blot and quantification of total protein extracts after ubiquitination inhibition with TAK‐243 (5 μM 12 h). Error bars correspond to the standard deviation of three biological replicates.

To prove this hypothesis, we used immunoprecipitation of ubiquitinated proteins and chemical inhibition of the ubiquitination pathway. For the first approach, we transfected HEK293T cells with a plasmid encoding ubiquitin with a histidine tag. The expression of a His‐tagged ubiquitin allowed the immunoprecipitation of ubiquitinated proteins, which could be detected by Western blot. First, we confirmed that H1 subtypes accumulated in HEK293T cells upon proteasome inhibition. The H1 complement in HEK293T is composed of five subtypes: H1.0, H1.2‐H1.4, and H1X. Like in T47D cells, all subtypes increased after treatment with MG132, with H1.0 and H1X being those more affected (Figure 5a,b).

After immunoprecipitating the protein extract with an antibody against the His‐tag, we confirmed by Western blot the presence of ubiquitinated β‐catenin in the cells treated with the inhibitor (Figure 5c). We also analyzed the more abundant subtypes expressed in HEK293T, H1.2‐H1.4. All three subtypes were immunoprecipitated due to unspecific interactions with the His‐tag antibody, as the main band detected corresponded to the unmodified protein. We also detected a faint band of higher molecular weight in H1.3 and H1.4. This band was present in the untreated and treated cells showing little or no increase after treatment with MG132 (Figure 5c). However, the increase in the molecular weight of approximately 15–20 kDa without intermediate bands hints that these bands may arise from the unspecific binding of the antibodies or, more likely, from protein aggregates containing H1 subtypes.

As a second approach, we used TAK‐243 to inhibit the ubiquitin‐activating enzyme (UBA1), the first step of the ubiquitination pathway (Figure 5d,e). After treatment, we found that β‐catenin, our positive control, accumulated more than two‐fold. Histone H1 subtypes maintained their levels, with ratios between 1.1 and 0.9 when compared to the untreated cells. The same effect was observed in the replication‐independent and two of the replication‐dependent subtypes in HeLa and T47D (Figure S10). Overall, our results indicate that the degradation of H1 subtypes is not dependent on ubiquitination.

Ubiquitin‐independent proteasomal degradation can be performed by the 20S proteasome directly or in association with regulatory subunits PA200 or PA28 (Fricker, 2020). The 20S proteasome can directly target basic proteins containing intrinsically disordered domains (Kudriaeva et al., 2019). Histone H1 is enriched in basic residues and contains two intrinsically disordered domains, so we analyzed whether the 20S proteasome catalyzed H1 degradation in vitro.

**FIGURE 6:**
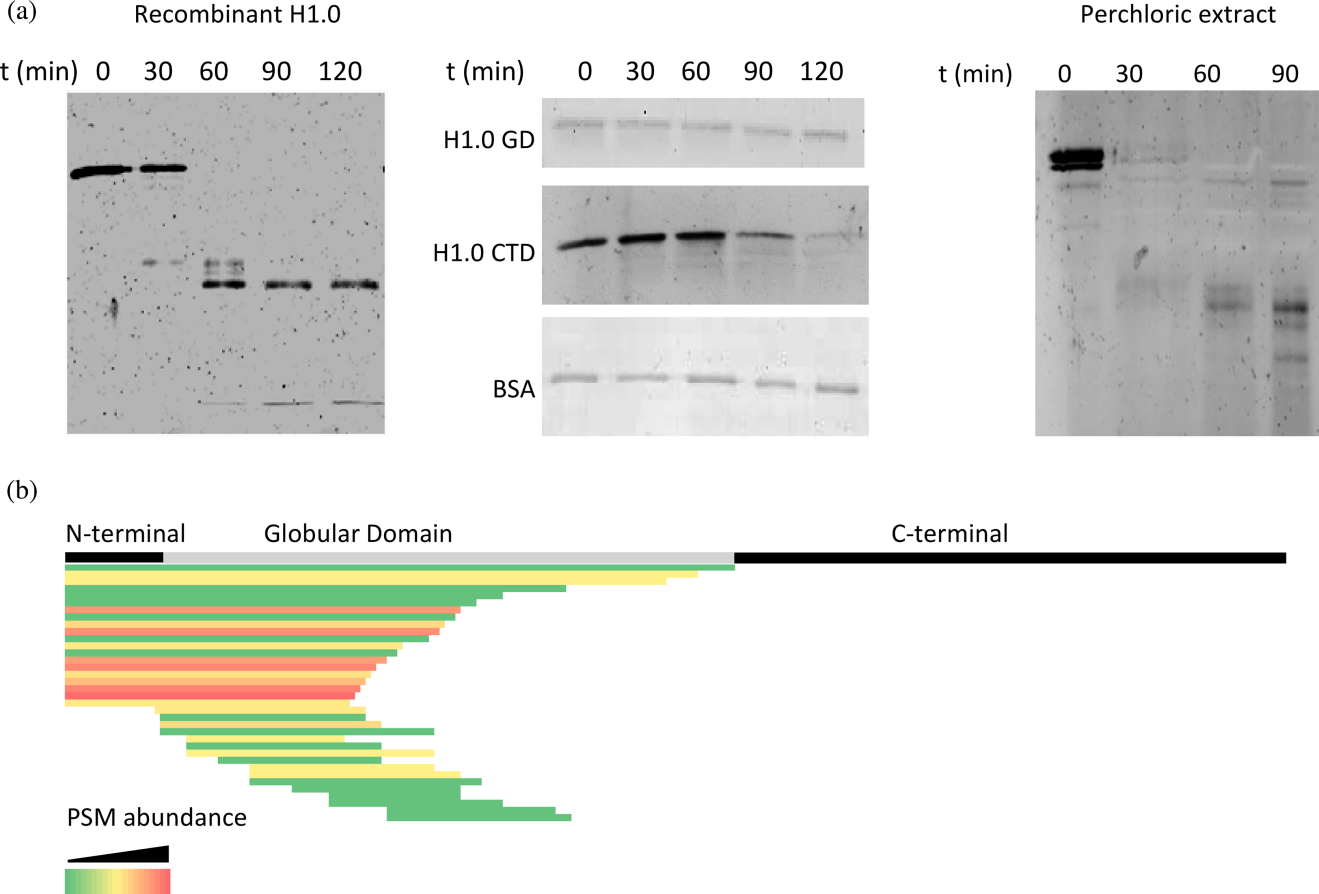
Histone H1 degradation by the 20S proteasome. (a) Silver staining of *in vitro* degradation of purified proteins with the 20S proteasome. H1.0, its C‐terminal domain (CTD), and its globular domain (GD) are recombinant proteins. Native H1s were obtained by perchloric extraction of T47D cells nuclei. Bovine serum albumin (BSA) was used as a negative control. (b) Schematic representation of the proteoforms of recombinant H1.0 identified by top‐down mass spectrometry after partial digestion with the 20S proteasome. Color scale corresponds to the number of protein‐spectrum matchs (PSMs) of each proteoform.

Using recombinant H1.0 as a model, we digested the whole protein and its structural domains with the 20S proteasome and analyzed the products by SDS‐PAGE. The whole protein was readily digested, with the intact protein disappearing after 60 min and detecting lower molecular weight degradation intermediates (Figure 6a). These results suggested that H1 could be degraded directly by the 20S proteasome. Furthermore, degradation of H1.0 was prevented by adding MG132 to the reaction, confirming that the digestion products were the result of the 20S proteasome activity (Figure S11).

We analyzed the contribution of H1 structural domains to the degradation by the 20S proteasome. The CTD intact band decreased with time, and products of lower molecular weight could be observed, showing similar behavior to the whole protein. In contrast, the GD and bovine serum albumin (BSA) used as a negative control remained stable throughout the reaction (Figure 6a). The NTD was not analyzed because even though it is also intrinsically disordered has only 20 residues, and therefore, it is not suitable for analysis with SDS‐PAGE. The whole H1.0, as well as the CTD, is expressed with a histidine tag for purification. To discount the possibility that the tag could be involved in targeting the 20S proteasome, we digested *in vitro* a mixture of native H1s extracted with perchloric acid from T47D. We observed a similar pattern to that of recombinant H1.0, indicating that the degradation of the protein by the 20S proteasome was not associated with the histidine tag.

To further characterize the contribution of the individual domains of H1.0 to the degradation by the 20S proteasome, we analyzed the digestion intermediates by mass spectrometry (Figure 6b, Table S1). The top‐down analysis of partially digested H1.0 allowed the identification of long peptides (>25 residues), detecting 39 proteoforms of the digested protein with more than one protein‐spectrum match (PSM). They could be divided into two groups. The first group had 23 proteoforms, 150 PSMs, and lacked the CTD. The second group had 16 proteoforms with 42 PSMs, lacking the CTD and the NTD. Almost all proteoforms of the second group had two or three PSMs, indicating their low abundance in the sample. These results indicate that the degradation of histone H1 by the 20S proteasome is determined by the intrinsically disordered properties of its CTD.

**FIGURE 7:**
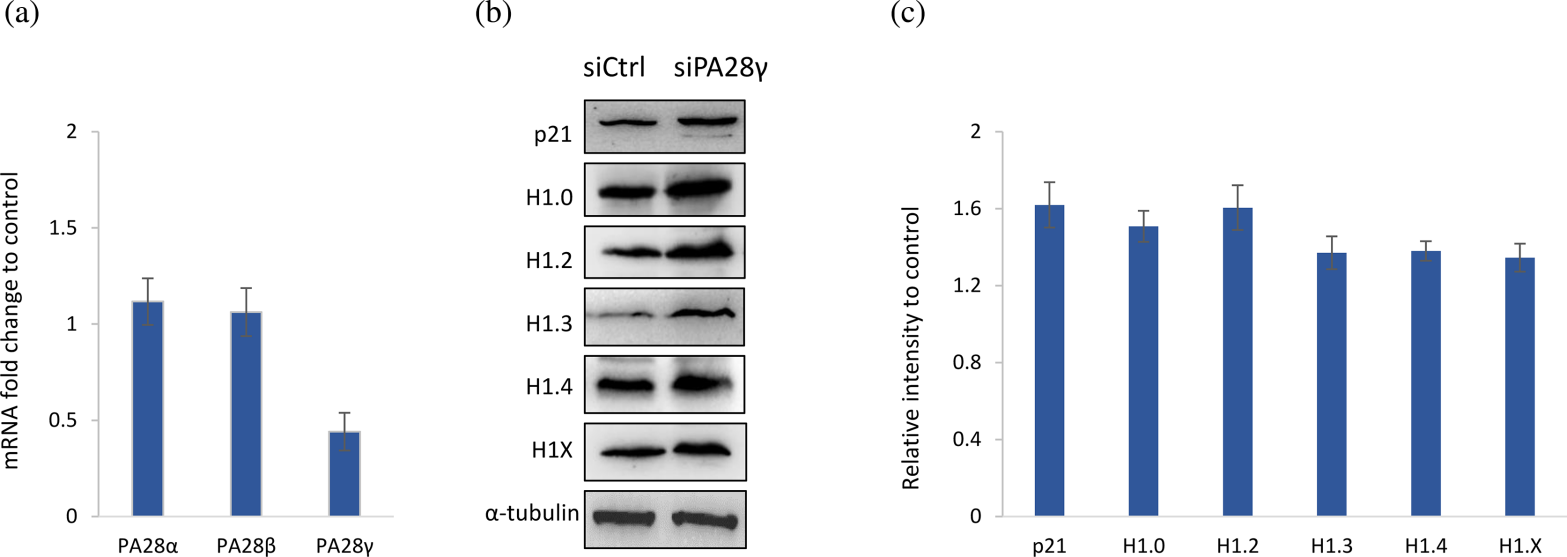
Role of PA28γ in the degradation of H1 subtypes. (a) Fold change of the transcript levels of the members of the PA28 family after transfection of PA28γ siRNA compared to control siRNA in HEK293T cells. (b) Western Blot of total protein extracts after PA28γ depletion. (c) Quantification of the Western blot images of three biological replicates. Error bars correspond to the standard deviation.

Several regulatory subunits facilitate the gate opening of the 20S proteasome for ubiquitin‐independent degradation. In particular, PA28γ promotes Ub‐independent degradation of other proteins with intrinsically disordered regions, such as p21 and the myelin basic protein (MBP) (Kudriaeva et al., 2019; Li et al., 2007) We examined the contribution of PA28γ to the degradation of H1 subtypes by siRNA transient knockdown and Western blot. After transfection, the siRNA targeting PA28γ reduced its mRNA to approximately 40% compared with the control siRNA but did not alter the other family members, PA28α and PA28β (Figure 7a). Depletion of PA28γ increased the protein levels of the positive control p21 by 1.6‐fold and of all H1 subtypes between 1.35‐ and 1.6‐fold (Figure 7b,c). These results suggest that PA28γ promotes H1 degradation by the 20S proteasome.

## 3 DISCUSSION

We have examined the role of the proteasome in the control of histone H1 protein levels. Inhibition of the proteasome with MG132 caused the accumulation of all H1 subtypes in several human cell lines, indicating that it is involved in H1 proteolysis. We observed a negative correlation between H1 accumulation and protein stability. The replication‐independent subtypes, especially H1.0, had higher accumulation and were less stable. The regulation of these subtypes at the protein level allows for fast changes in the H1 complement in response to different stimuli. Experimental evidence has confirmed that H1.0 expression is regulated by external stimuli and that this subtype is a key player in the compensation of the H1 levels upon the knockdown of individual or multiple subtypes (Di Liegro et al., 2018; Izquierdo‐Bouldstridge et al., 2017; Millán‐Ariño et al., 2016; Pan & Fan, 2016).

MG132 inhibits the proteasome and cellular proteases like calpains (Fricker, 2020). For that reason, we analyzed H1 accumulation in the presence of BTZ, a specific inhibitor of the proteasome (Fricker, 2020; Goldberg, 2012; Kisselev et al., 2012). We found that H1 accumulated less than in MG132, with some changes in specific subtypes. Two explanations, alone or together, could account for our findings: the differences between the two inhibitors and the action of other proteolytic mechanisms. Regarding the first possibility, MG132 at the doses used in our experiments inhibits all three catalytic activities in the proteasome (Bibo‐Verdugo et al., 2017). Meanwhile, BTZ preferentially inhibits the chymotrypsin‐like activity, to a lesser extent, the post‐glutamyl activity, and does not inhibit the trypsin‐like activity in the standard proteasome (Kisselev et al., 2012). Considering that histone H1 is enriched in basic amino acids, the trypsin‐like activity could be essential for its degradation, explaining some of the differences observed between the inhibitors. As for the second possibility, further experiments will determine whether calpains or other proteases contribute to H1 degradation.

Upon proteasome inhibition with MG132, the transcript levels of H1 subtypes decreased. This difference was not associated with significant changes in the cell cycle. Although the indirect effects of the drug cannot be ruled out, our results open the possibility that transcriptional downregulation could also contribute to preventing the excess of H1 subtypes. The transcriptional regulation of H1 subtypes during the cell cycle and the evidence of co‐transcriptional regulation are in favor of this feedback loop (Duronio & Marzluff, 2017; Ponte et al., 2021). This result suggests that the control of the protein levels of histone H1 is a process with multiple layers.

The multilayered regulation of H1 subtypes at the protein level could be necessary to protect the cell from the cytotoxic effect of its accumulation. An excess of histone H1 can result in its unspecific binding to chromatin, which can alter chromatin structure and transcription. Local alterations in chromatin structure, as well as in the transcript levels were observed in yeast after overexpression of core histones (Singh et al., 2010). In the case of histone H1, some biological systems, such as chicken erythrocytes confirm that a higher content of H1 in the nucleus promotes chromatin compaction and transcriptional silencing (Beacon & Davie, 2021; Delcuve & Davie, 1989). Inducible overexpression of recombinant H1.0 and H1.2 in mouse fibroblasts led to a reduction in the protein levels of the rest of the H1 subtypes while producing a moderate increase in the H1: nucleosome ratio up to 1.3 when H1.0 was overexpressed (Brown et al., 1996). H1 overexpression reduced chromatin nuclease accessibility, increased nucleosome spacing, and caused alterations in gene expression (Bhan et al., 2008; Gunjan et al., 1999).

Histone H1 is mainly a nuclear protein, but a cytoplasmatic pool of H1 amounting to approximately 10% has been described (Zlatanova et al., 1990). Upon proteasome inhibition, we observed an increase in the cytoplasmatic pool of all H1 subtypes. The accumulation of histone H1 in the cytosol has been observed in mouse models of prion and Alzheimer's diseases (Bolton & Betmouni, 1999) and in response to the CDK inhibitor flavopiridol in primary chronic lymphoid leukemia cells (Harshman et al., 2013). The preferential accumulation of histone H1 in the cytoplasm, maintaining the H1: nucleosome ratio almost constant in the nucleus, prevented global changes in chromatin structure, as observed in the nuclease accessibility assay. However, local alterations are still possible, as we detected rearrangements in the proportions of H1 subtypes bound to chromatin. Changes in the nuclear chromatin‐bound H1 complement and the proportions of H1 subtypes in the cytoplasm could result from the combined effect of their differential characteristics, including protein stability, nuclear localization, chromatin affinity, and PTMs (Andrés et al., 2020; Izquierdo‐Bouldstridge et al., 2017; Kumar et al., 2023; Millán‐Ariño et al., 2016). In addition, the accumulation of H1 in the cytoplasm could cause unspecific binding to RNA molecules, potentially altering RNA‐associated functions. The propensity of H1 to bind RNA molecules and promote phase separation has been recently reported, although its relevance in our conditions was not explored (Leicher et al., 2022).

It is known that phosphorylation of H1 promotes its accumulation in the cytoplasm (Bleher & Martin, 1999). We found a slight increase in phosphorylated H1 in the cytoplasm, suggesting that this modification could contribute to its accumulation in this compartment. The extent of the contribution of phosphorylation could be higher than the one we observed, considering that the available antibodies didn't target directly phosphorylated positions in H1.5, the subtype with the greatest increase in the cytoplasm. Other PTMs that decrease H1 affinity for chromatin like acetylation or parylation, together with phosphorylation at other positions could also play a role in H1 localization to the cytoplasm (Andrés et al., 2020; Kumar et al., 2023). However, it is reasonable to think that a part of the H1 in the cytoplasm corresponds to newly synthesized proteins that are not transported to the nucleus.

The ubiquitin‐proteasome system controls the metabolism of more than half of the intracellular proteins (Ciechanover, 2015). This pathway controls the protein levels of some core histone variants in response to EGF stimulation, SIRT1 overexpression, and during mitosis in embryonic cells (Baptista et al., 2013; Oh et al., 2020; Xia et al., 2017). In contrast, the accumulation of H1 subtypes upon proteasome inhibition didn't result in the appearance of high molecular weight bands corresponding to monoubiquitinated or polyubiquitinated H1s. Immunoprecipitation of His‐tagged ubiquitin showed that the contribution of this proteolytic mechanism to the control of H1 subtypes is almost nonexistent. Chemical inhibition of the first step of the ubiquitin pathway did not affect the levels of H1 subtypes, confirming that their degradation is independent of ubiquitination. However, ubiquitinated forms of H1.2, H1.4, H1.0, and H1X were found in nuclear proteasome‐containing foci induced by acute hyperosmotic stress (Yasuda et al., 2020). This finding suggests that the Ub‐independent pathway may be responsible for H1 degradation under normal conditions, while the Ub‐dependent pathway may also contribute to H1 degradation under certain stress conditions.

Histone H1 has already been described to be degraded by the 20S proteasome in response to oxidative stress (Cervantes‐Laurean et al., 2005; Ullrich & Grune, 2001). However, the degron for its recognition by 20S or the proteolytic mechanisms controlling its degradation in normal conditions have not been described. One of the signals for protein targeting to 20S proteasome involves intrinsically disordered regions enriched in basic amino acids (Kudriaeva et al., 2019). We found that 20S proteasome can degrade directly recombinant and endogenous H1 subtypes, as well as its CTD. Proteomic analysis of partially digested H1 showed the presence of long peptides lacking the CTD, suggesting that this domain acts as a direct proteasome signal. These results suggest that this mechanism is involved in the targeting and degradation of H1 in normal conditions. Moreover, the increase of the levels of the 20S in certain stress conditions could promote the degradation of damaged H1, among other proteins, preventing chromatin proteotoxicity (Sahu et al., 2021).

It has been described that intrinsically disordered regions bind to the 20S proteasome, sometimes aided by the presence of basic residues (Kudriaeva et al., 2019; Myers et al., 2018; Sahu et al., 2021). Cryo‐EM experiments have shown that the initial binding induced structural alterations consistent with the opening of the central pore in the α‐ring and the entering of the substrate to the proteolytic chamber (Sahu et al., 2021). The opening in the α‐ring was wide enough to allow the entry of partially unfolded small globular proteins like ubiquitin and DHFR, when fused to IDR long enough to reach the proteolytic chamber that provided the pulling force for the degradation of the rest of the chimeric protein (Kudriaeva et al., 2019; Sahu et al., 2021). In the case of H1, its long intrinsically disordered CTD may serve as an initiation site for the recognition by the 20S proteasome with its evenly distributed lysine residues playing an important role in the initial interaction (Kudriaeva et al., 2019). Binding of the CTD would induce the opening of the gate of the 20S proteasome, allowing the entry and degradation of the rest of the protein.

Within the cells, Ub‐independent proteasomal degradation can be carried out by the 20S proteasome alone or aided by the regulatory subunits PA28 and PA200. Among this group, PA28γ is ubiquitously expressed and mainly localized in the nucleus. It forms a heptameric regulatory cap that facilitates gate opening of the 20S proteasome and enhances its proteolytic activity (Cascio, 2021). PA28γ is involved in the degradation of several proteins with intrinsically disordered domains, including some cell cycle regulators, such as p21, p16, and p19 (Chen et al., 2007; Li et al., 2007). Partial depletion of PA28γ caused an accumulation of H1 subtypes, suggesting that this regulatory subunit promotes their degradation. Our results show that cellular proteins composed of intrinsically disordered and stably folded domains can be completely degraded by the 20S proteasome alone or coupled with PA28γ, highlighting the importance of this proteolytic pathway in proteostasis.

## 4 CONCLUSIONS

Histone H1 protein levels are tightly regulated by proteasome degradation and, probably, by transcriptional downregulation. Upon proteasome inhibition, the total amount of H1 within the nucleus remained unchanged, while its excess accumulated in the cytoplasm. The alteration in the subcellular distribution minimized the changes in chromatin structure and transcription that could arise from H1 accumulation within the nucleus. We described, for the first time, that H1 intrinsically disordered CTD acts as a primary determinant for its targeting and degradation by the 20S proteasome, a process enhanced by the regulatory subunit PA28γ.

## 5 METHODS

### 5.1 Cell culture and treatments

All the cell lines were grown at 37°C and 5%CO_2_ in their specific culture media supplemented with 10% fetal calf serum (Gibco) and 1% penicillin–streptomycin (Ddbiolab). Human embryonic kidney 293 T cells (HEK293T) and cervical carcinoma cells (HeLa) were cultured in DMEM Glutamax (Corning). For human breast cancer cells (T47D), the culture media was RPMI (Corning), supplemented with 2 mM L‐glutamine (Ddbiolab). After harvesting, cells were counted in an automated cell counter (Bio‐Rad). For proteasome inhibition, cells were treated with 20 μM MG132 (Sigma) or 20 nM bortezomib (BTZ) (Labnet) for 12 h. Both drugs were dissolved in dimethyl sulfoxide (DMSO). For translation inhibition, cells were treated with 10 μg/mL cycloheximide (Sigma) for 8 h. For simultaneous inhibition of the proteasome and protein translation, cells were treated with MG132 and cycloheximide at the concentrations described above for 8 h. For ubiquitination inhibition, cells were treated with 5 μM TAK‐243 (MLN7243) (CliniSciences) for 12 h.

### 5.2 Analysis of proteasomal activity

Proteasome inhibition was assessed by measuring the chymotrypsin‐like activity using the fluorogenic peptide, Suc‐LLVY‐AMC, as described by (Zafar et al., 2007). Fluorescence scans were recorded in a Varian Cary Eclipse fluorimeter using excitation wavelength, 345 nm, and emission wavelength interval, 360–500 nm. As a positive control, Suc‐LLVY‐AMC was digested *in vitro* with 1 unit of chymotrypsin for 15 min at 37°C. The chymotrypsin activity was calculated using the fluorescence values at 438 nm and expressed as a percentage of the Suc‐LLVY‐AMC digested *in vitro.*

### 5.3 Preparation of protein extracts

Cells were harvested by trypsinization, washed twice with phosphate buffered saline (PBS). For preparing total protein extracts, cells were resuspended in a 50 mM Tris pH 8 buffer containing 300 mM NaCl, 1% Triton X‐100, 0.5% sodium deoxycholate, 3 mM DTT, and a protease inhibitor cocktail (PIC), and incubated for 30 min on ice. The mixture was centrifugated at 16,000× *g*, 30 min, at 4°C. The suspension was passed through a needle until it was homogeneous, and centrifugated at 16,000× *g* 30 min, at 4°C. The supernatant contained a mixture of cellular proteins.

For obtaining cytoplasmatic and nuclear fractions, cells were resuspended in 1 0 mM HEPES pH 7.9, 1.5 mM MgCl_2_, 10 mM KCl, and PMSF 0.1 mM. Cell membrane was lysed in a Dounce homogenizer. The nuclei were pelleted by centrifugation at 800 *g* for 5 min. The supernatant, while containing other H1‐free organelles, was enriched in cytoplasmatic proteins.

For obtaining linker or total histones acid extraction was used. Perchloric extraction of linker histones in the nuclear fraction was performed as previously described (Sarg et al., 2015). Histone sulfuric extraction of whole cells or the nuclear fraction was performed using 0.2 M sulfuric acid, instead of perchloric acid with the same procedure.

All the protein extracts were analyzed by SDS‐PAGE and stored at −20°C. Protein concentration was determined with Bradford.

### 5.4 Western blot

Equivalent amounts of total proteins (10 μg) were separated in a 12% denaturing polyacrylamide gel electrophoresis (SDS‐PAGE) and electrotransferred to a polyvinylidene difluoride membrane (PVDF) (EMD Millipore) at 100 V for 1 h. Immunoblot analyses were performed with the conditions recommended by the manufacturer for the primary and secondary antibodies (Table S2). Specificity of primary antibodies have been validated using knock‐down cell lines (Serna‐Pujol et al., 2022). Blots were visualized with Clarity Western ECL substrate (Bio‐Rad) in a Chemidoc imaging system (Bio‐Rad). Band intensities were quantified using Image Lab software (Bio‐Rad). α‐tubulin or histone H1 were used as loading control.

### 5.5 RT‐qPCR

Total RNA was purified from one million cells with the High Pure RNA Isolation kit (Roche) following the manufacturer's instructions. Purified RNA was quantified by Nanodrop (Thermo Scientific), and 100 ng were retrotranscribed with iScriptTM cDNA synthesis kit (Bio‐Rad) using random hexamers as primers. H1 subtypes and GAPDH were amplified by qPCR using primers specific (Table S3). Fold change was calculated using the ddCT method (Livak & Schmittgen, 2001).

### 5.6 Immunofluorescence

Cells were grown on the desired conditions, counted, and diluted in PBS to a final concentration of 5·10^4^ cells/mL. They were spun down for 10 min at 500× *g* in a Thermo Shandon Cytospin 3 using a single‐chamber Cytospin funnel as described by Izquierdo‐Bouldstridge et al. (2017). Cells were air‐dried for 1 h at room temperature (RT) and then fixed with 2% paraformaldehyde at RT for 10 min. Fixed cells were permeabilized with PBS‐0.5% Triton X‐100 and blocked with PBS 0.1% Tween, 2% bovine serum albumin for 1 h at RT. For immunodetection, primary antibodies were incubated at 4°C overnight and secondary antibodies at RT for 1 h (Table S2). Nuclei were stained with 4′,6‐diamidino‐2‐fenilindol (DAPI) at 0.1 μg/mL. Images were taken using a Leica SP5 AOBS confocal microscope. Two images per slide of three biological replicates, amounting 35–70 cells per condition, were analyzed with Imaris for Cell Biologist package (Oxford instruments). This application allows fluorescence quantification within predefined 3D surfaces. For each cell the fluorescence intensity of H1 was quantified in the 3D surface of the whole cell and that of the nucleus. The nuclear surface was determined using DAPI as a marker. A relative fluorescence score for cytoplasmatic H1 was calculated using the difference between total and nuclear fluorescence of individual cells, normalizing the acquisition parameters for the measurements to be comparable. All cells were visually evaluated to discard artifacts. The values were expressed as a percentage of the total fluorescence. Kolmogorov–Smirnov test was used to determine the adjustment of the values to a normal distribution. The differences between the samples were assessed with the Student's *T*‐test. All the statistical analyses were performed at https://www.socscistatistics.com/.

### 5.7 Chromatin extraction

To study H1 subtypes bound to chromatin in untreated cells and after treatment with MG132, chromatin fragments were prepared by sonication. Cells were fixed using 1% formaldehyde, harvested, and sonicated in a Bioruptor (Diagenode) to generate chromatin fragments between 200 and 500 bp. The cross‐linking was reversed by incubation at 65°C overnight, and the proteins were analyzed by Western blot.

### 5.8 Nuclease accessibility assay

The nuclear pellet, obtained as described above, was washed in a buffer containing 10 mM Tris pH 7.4, 3 mM MgCl_2_, 60 mM KCl, and 1% thiodiglycol, centrifuged at 800× *g* for 5 min, and resuspended in the same buffer to a final DNA concentration of 1 mg/mL. Chromatin digestion was performed by adding 0.5 mM CaCl_2_ and micrococcal nuclease (Sigma) at a concentration of 1 U/50 μg of DNA at 37°C. Digestion was stopped adding EDTA up to 10 mM. Nuclei were sedimented at 4500× *g* for 5 min and lysed in Tris‐EDTA buffer (TE) pH 8.0 for 30 min at 4°C. Soluble chromatin was obtained after centrifugation at 16,000× *g* for 10 min. Proteins in the supernatant were digested with 100 μg/mL proteinase K (Sigma) at 37°C overnight. DNA fragments were extracted with phenol‐chloroform‐isoamyl alcohol (25:24:1) and analyzed by agarose gel electrophoresis.

### 5.9 Analysis of ubiquitinated proteins

Transfection of HEK293T cells with pHis‐Ubiquitin (Addgene, 31815) was performed by the calcium phosphate‐DNA precipitation method (Kwon & Firestein, 2013). After 48 h, cells were treated with MG132, harvested, and total protein extracts were prepared as described above. Ubiquitinated proteins were immunoprecipitated using magnetic Protein A Dynabeads (Thermofisher) loaded with an antibody against His‐tagged proteins (Finetest, FNab00008). Immunoprecipitation was carried out overnight, at 4°C, in 20 mM Tris pH 7.5, 150 mM NaCl, 1 mM EDTA, 1 mM EGTA, 1% Triton X‐100, and (PIC) (Thermofisher). Beads were washed three times, and proteins were eluted in 2× SDS sample buffer at 95°C for 10 min. The presence of ubiquitinated H1s was analyzed by Western blot, using β‐catenin as a positive control.

### 5.10 Transient PA28γ knockdown

HEK293T cells were transfected with 9 nM of PA28γ custom siRNA (5′‐GAAUCAAUAUGUCACUCUA‐3′) (Eurofins) or a negative control siRNA (1,022,076, Qiagen) using METAFECTENE SI+ (Biontex). After 48 h, cells were harvested, and total protein extracts were prepared as described above. The effectivity of the siRNA depletion was analyzed by RT‐qPCR (Table S3). The effect of the partial depletion on the protein levels was analyzed by Western blot, using p21 as a positive control.

### 5.11 Preparation of recombinant proteins

Recombinant proteins corresponding to H1.0, its globular and C‐terminal domains were expressed and purified from *Escherichia coli*, as previously described (Roque et al., 2004).

### 5.12 In vitro digestion with the 20S proteasome

We analyzed the degradation kinetics of 200 ng/condition of recombinant H1.0, its GD and CTD, and 1 μg/condition of the perchloric acid purified histone mixture from T47D cells, using the protocol described by Kudriaeva et al. (2019). Bovine serum albumin (Sigma) was used as a negative control. Proteolytic digestion was performed in 20 mM Tris pH 7,5, 20 mM NaCl, 1 mM EDTA, and 1 mM DTT, in the presence of 5 nM of 20S proteasome (Boston Biochem, E‐360), at 37°C. The reaction was stopped by adding electrophoresis loading buffer and incubating the samples at 95°C for 5 min. The digestion products were analyzed by 12%–15% SDS‐PAGE and stained with Coomassie blue or silver (Silver stain plus kit, Bio‐rad). Images were taken using a Chemidoc imaging system (Bio‐Rad). Degradation intermediates were characterized by top‐down mass spectrometry.

### 5.13 Mass spectrometry analysis

Degradation intermediates of H1.0 digested with proteasome 20S *in vitro* were analyzed by top‐down proteomics at the IRB Barcelona Mass Spectrometry and Proteomics Core Facility. The sample was cleaned‐up using C4‐tips (polyLC) and separated by nanoLC with an Acquity UPLC M‐Class BioResolve mAb Column (Waters). Proteoforms were eluted using a combination of two eluents: A. H_2_O 0.1% formic acid; B. CH_3_CN 0.1% formic acid in the following proportions: 10% to 50% of B in 120 min + 50% to 85% in 7 min. The chromatography was performed at a flow rate of 300 nL/min at 60°C. The sample was ionized by nESI, using Advion Triversa Nanomate (Advion BioSciences) as a source at 1.7 kV, 0.5 psi, in a positive mode. MS/MS was performed in an Orbitrap Fusion Lumos™ Tribrid (Thermo Scientific) in a data‐dependent mode. The ions were fragmented by ETD, with 10% supplemental energy from HCD. Proteoform identification was performed using Top‐Down PSCW database creation from XML inside Proteome Discoverer software (v2.5) (Thermo Scientific). BioPharma Finder v4.0 (Thermo Fisher Scientific) was used to extract averaged mass spectra from detected chromatographic peaks (MS1). Deconvolution was done using the auto Xtract algorithm on resolved m/z charged species. The slice window option was set to a target average spectrum width of 0.1 min. The recombinant H1.0 sequence was introduced to find a match between the experimental and theoretical masses.

## AUTHOR CONTRIBUTIONS

**A. Roque:** Conceptualization; investigation; writing – original draft; writing – review and editing; formal analysis; supervision; resources; funding acquisition. **D. García‐Gomis:** Investigation; formal analysis. **J. López:** Investigation; formal analysis. **A. Calderón:** Investigation. **M. Andrés:** Investigation. **I. Ponte:** Investigation; conceptualization; supervision; writing – original draft; formal analysis; writing – review and editing.

## Supporting information


**FIGURE S1.** Inhibition of chymotrypsin activity after treatment with MG132 and BTZ. (a) Fluorescence spectra of Suc‐LLVY‐AMC peptide in solution (continuous line) and digested with chymotrypsin *in vitro* (dashed line). (b) Percentage of chymotrypsin activity in three biological replicates of T47D cells grown in presence of DMSO, 20 μM MG132 (MG132), and 20 nM Bortezomib (BTZ) for 12 h. The results are expressed as a percentage of the fluorescence emitted at 438 nm by Suc‐LLVY‐AMC peptide digested with chymotrypsin *in vitro*. Error bars correspond to the standard deviation.
**FIGURE S2.** Accumulation of histone H1 subtypes upon proteasome inhibition in HeLa. (a) Western blot of H1 subtypes after treatment MG132, as described in materials and methods. (b) Quantification of the Western blot results in three biological replicates normalized by tubulin. Error bars correspond to the standard deviation.
**FIGURE S3.** Inhibition of the proteasome and protein synthesis in T47D. Western blot of representative H1 subtypes after treatment with MG132 and cycloheximide, as described in materials and methods. Quantification of the western blot images in three biological replicates normalized by tubulin. Error bars correspond to the standard deviation.
**FIGURE S4.** Protein stability of H1 subtypes in HeLa. (a) Western blot of H1 subtypes after treatment cycloheximide (CHX), as described in materials and methods. (b) Protein fraction in two biological replicates of each protein remaining after 8 h of treatment with CHX. Error bars correspond to the standard deviation. (c) Correlation between the accumulation after MG132 treatment and protein stability. r, correlation coefficient.
**FIGURE S5.** Changes in the transcript levels of H1 subtypes after proteasome inhibition. Cells were treated with MG132, as described in materials and methods. RT‐qPCR results are expressed as fold change of the relative expression to GAPDH of each transcript in MG132 treated cells respect to the control cells grown in media supplemented with 0.2% DMSO of three biological replicates. (a) T47D. (b) HeLa. Error bars represent the standard deviation.
**FIGURE S6.** Analysis of cell cycle after proteasome inhibition in T47D. Flux cytometry profiling of cells stained with propidium iodine. (a) Control cells grown in 0.2% DMSO. (b) Cells treated with MG132, as described in material and methods. (c) Proportion of cells in each phase in two biological replicates, expressed as percentages. (d) Percentage of live cells after 12 h in the presence of DMSO and MG132. Error bars correspond to the standard deviation.
**FIGURE S7.** Accumulation of histone H1 subtypes in the cytoplasm after proteasome inhibition. Western Blots of a cytoplasmatic protein extract of T47D cells after treatment with MG132 (20 μM 12 h).
**FIGURE S8.** Accumulation of histone H1 subtypes in the cytoplasm of T47D cells after proteasome inhibition with Bortezomib. (a) Representative immunofluorescence images of H1 somatic subtypes in T47D cells. Cells were treated with DMSO and Bortezomib (20 nM in DMSO, 12 h). Cellular nuclei were stained with DAPI. (b) Box plots correspond to the quantification of 35–70 cells/variants and condition. Asterisks denote the *p*‐value of the two‐tailed Student's *t*‐test showing the significance of the difference between untreated and treated cells *p‐value <0.05; **p‐value <0.01; ***p‐value <0.001; n.s, not significant.
**FIGURE S9.** Accumulation of histone H1.2 in the cytoplasm of HeLa cells after proteasome inhibition. (a) Representative immunofluorescence images of H1.2 in HeLa cells. Cells were treated with DMSO, MG132 (20 μM), and Bortezomib (20 nM) 12 h. Cellular nuclei were stained with DAPI. (b) Box plots correspond to the quantification of 35–70 cells/variants and condition. Asterisks denote the p‐value of the two‐tailed Student's *t*‐test showing the significance of the difference between untreated and treated cells *p‐value <0.05; **p‐value <0.01; ***p‐value <0.001; n.s, not significant.
**FIGURE S10.** Inhibition of the ubiquitin pathway in human cell lines. Western blot images of T47D (a) and HeLa (b) cells treated with 5 μM TAK‐243 for 12 h. Quantification of the western blot images in three biological replicates normalized by tubulin in T47D (c) and HeLa (d). Error bars correspond to the standard deviation.
**FIGURE S11.** Inhibition of H1.0 degradation by the 20S proteasome in the presence of MG132. Silver staining of 15% SDS‐PAGE of recombinant H1.0 incubated with the 20S proteasome and MG132 (20 μM).

**TABLE S1.** Proteoforms of H1.0 digested with the 20S proteasome *in vitro*.

**TABLE S2.** Antibodies used for Western blot (WB) and immunofluorescence (IF).

**TABLE S3.** Sequence of the primers and conditions used for quantitative PCR.

## References

[pro4970-bib-0001] Aberle H , Bauer A , Stappert J , Kispert A , Kemler R . β‐Catenin is a target for the ubiquitin–proteasome pathway. EMBO J. 1997;16:3797–3804. 9233789 10.1093/emboj/16.13.3797 PMC1170003

[pro4970-bib-0002] Andrés M , García‐Gomis D , Ponte I , Suau P , Roque A . Histone H1 post‐translational modifications: update and future perspectives. IJMS. 2020;21:5941. 32824860 10.3390/ijms21165941 PMC7460583

[pro4970-bib-0003] Baptista T , Graça I , Sousa EJ , Oliveira AI , Costa NR , Costa‐Pinheiro P , et al. Regulation of histone H2A.Z expression is mediated by sirtuin 1 in prostate cancer. Oncotarget. 2013;4:1673–1685. 24127549 10.18632/oncotarget.1237 PMC3858554

[pro4970-bib-0004] Bates DL , Thomas JO . Histories H1 and H5: one or two molecules per nucleosome? Nucleic Acids Res. 1981;9:5883–5894. 7312631 10.1093/nar/9.22.5883 PMC327571

[pro4970-bib-0005] Beacon TH , Davie JR . Transcriptionally active chromatin—lessons learned from the chicken erythrocyte chromatin fractionation. Cells. 2021;10:1354. 34070759 10.3390/cells10061354 PMC8226759

[pro4970-bib-0006] Bhan S , May W , Warren SL , Sittman DB . Global gene expression analysis reveals specific and redundant roles for H1 variants, H1c and H10, in gene expression regulation. Gene. 2008;414:10–18. 18372120 10.1016/j.gene.2008.01.025 PMC2706510

[pro4970-bib-0007] Bibo‐Verdugo B , Jiang Z , Caffrey CR , O'Donoghue AJ . Targeting proteasomes in infectious organisms to combat disease. FEBS J. 2017;284:1503–1517. 28122162 10.1111/febs.14029

[pro4970-bib-0008] Bleher R , Martin R . Nucleo‐cytoplasmic translocation of histone H1 during the HeLa cell cycle. Chromosoma. 1999;108:308–316. 10525967 10.1007/s004120050382

[pro4970-bib-0009] Bolton R‐C , Betmouni P . Non‐nuclear histone H1 is upregulated in neurones and astrocytes in prion and Alzheimer's diseases but not in acute neurodegeneration. Neuropathol Appl Neurobiol. 1999;25:425–432. 10564533 10.1046/j.1365-2990.1999.00171.x

[pro4970-bib-0010] Brown DT , Alexander BT , Sittman DB . Differential effect of H1 variant overexpression on cell cycle progression and gene expression. Nucleic Acids Res. 1996;24:486–493. 8602362 10.1093/nar/24.3.486 PMC145659

[pro4970-bib-0011] Cascio P . PA28γ: new insights on an ancient proteasome activator. Biomolecules. 2021;11:228. 33562807 10.3390/biom11020228 PMC7915322

[pro4970-bib-0012] Cerf C , Lippens G , Muyldermans S , Segers A , Ramakrishnan V , Wodak SJ , et al. Homo‐ and heteronuclear two‐dimensional NMR studies of the globular domain of histone H1: sequential assignment and secondary structure. Biochemistry. 1993;32:11345–11351. 8218199 10.1021/bi00093a011

[pro4970-bib-0013] Cervantes‐Laurean D , Roberts MJ , Jacobson EL , Jacobson MK . Nuclear proteasome activation and degradation of carboxymethylated histones in human keratinocytes following glyoxal treatment. Free Radic Biol Med. 2005;38:786–795. 15721989 10.1016/j.freeradbiomed.2004.11.030

[pro4970-bib-0014] Chen X , Barton LF , Chi Y , Clurman BE , Roberts JM . Ubiquitin‐independent degradation of cell‐cycle inhibitors by the REGγ proteasome. Mol Cell. 2007;26:843–852. 17588519 10.1016/j.molcel.2007.05.022 PMC2031223

[pro4970-bib-0015] Ciechanover A . The unravelling of the ubiquitin system. Nat Rev Mol Cell Biol. 2015;16:322–324. 25907614 10.1038/nrm3982

[pro4970-bib-0016] Delcuve GP , Davie JR . Chromatin structure of erythroid‐specific genes of immature and mature chicken erythrocytes. Biochem J. 1989;263:179–186. 2604693 10.1042/bj2630179 PMC1133406

[pro4970-bib-0017] Dhaenens M , Glibert P , Meert P , Vossaert L , Deforce D . Histone proteolysis: a proposal for categorization into ‘clipping’ and ‘degradation’: prospects & overviews. Bioessays. 2015;37:70–79. 25350939 10.1002/bies.201400118 PMC4305269

[pro4970-bib-0018] Di Liegro C , Schiera G , Di Liegro I . H1.0 linker histone as an epigenetic regulator of cell proliferation and differentiation. Genes. 2018;9:310. 29925815 10.3390/genes9060310 PMC6027317

[pro4970-bib-0019] Duerre JA , Lee CT . In VIVO methylation and turnover of rat BRAIN histones. J Neurochem. 1974;23:541–547. 4421616 10.1111/j.1471-4159.1974.tb06057.x

[pro4970-bib-0020] Duronio RJ , Marzluff WF . Coordinating cell cycle‐regulated histone gene expression through assembly and function of the histone locus body. RNA Biol. 2017;14:726–738. 28059623 10.1080/15476286.2016.1265198 PMC5519241

[pro4970-bib-0021] Fan Y , Nikitina T , Morin‐Kensicki EM , Zhao J , Magnuson TR , Woodcock CL , et al. H1 linker histones are essential for mouse development and affect nucleosome spacing in Vivo. Mol Cell Biol. 2003;23:4559–4572. 12808097 10.1128/MCB.23.13.4559-4572.2003 PMC164858

[pro4970-bib-0022] Fan Y , Nikitina T , Zhao J , Fleury TJ , Bhattacharyya R , Bouhassira EE , et al. Histone H1 depletion in mammals alters global chromatin structure but causes specific changes in gene regulation. Cell. 2005;123:1199–1212. 16377562 10.1016/j.cell.2005.10.028

[pro4970-bib-0023] Fricker LD . Proteasome inhibitor drugs. Annu Rev Pharmacol Toxicol. 2020;60:457–476. 31479618 10.1146/annurev-pharmtox-010919-023603

[pro4970-bib-0024] Goldberg AL . Development of proteasome inhibitors as research tools and cancer drugs. J Cell Biol. 2012;199:583–588. 23148232 10.1083/jcb.201210077 PMC3494858

[pro4970-bib-0025] Gunjan A , Alexander BT , Sittman DB , Brown DT . Effects of H1 histone variant overexpression on chromatin structure. J Biol Chem. 1999;274:37950–37956. 10608862 10.1074/jbc.274.53.37950

[pro4970-bib-0026] Hansen JC , Lu X , Ross ED , Woody RW . Intrinsic protein disorder, amino acid composition, and histone terminal domains. J Biol Chem. 2006;281:1853–1856. 16301309 10.1074/jbc.R500022200

[pro4970-bib-0027] Harshman SW , Chen MM , Branson OE , Jacob NK , Johnson AJ , Byrd JC , et al. Isolation and analysis of linker histones across cellular compartments. J Proteome. 2013;91:595–604. 10.1016/j.jprot.2013.08.022 PMC3863389 24013129

[pro4970-bib-0028] Hendzel MJ , Lever MA , Crawford E , Th'ng JPH . The C‐terminal domain is the primary determinant of histone H1 binding to chromatin in Vivo. J Biol Chem. 2004;279:20028–20034. 14985337 10.1074/jbc.M400070200

[pro4970-bib-0029] Izquierdo‐Bouldstridge A , Bustillos A , Bonet‐Costa C , Aribau‐Miralbés P , García‐Gomis D , Dabad M , et al. Histone H1 depletion triggers an interferon response in cancer cells via activation of heterochromatic repeats. Nucleic Acids Res. 2017;45:11622–11642. 28977426 10.1093/nar/gkx746 PMC5714221

[pro4970-bib-0030] Jiang T‐X , Ma S , Han X , Luo Z‐Y , Zhu Q‐Q , Chiba T , et al. Proteasome activator PA200 maintains stability of histone marks during transcription and aging. Theranostics. 2021;11:1458–1472. 33391545 10.7150/thno.48744 PMC7738882

[pro4970-bib-0031] Kisselev AF , van der Linden WA , Overkleeft HS . Proteasome inhibitors: an expanding Army attacking a unique target. Chem Biol. 2012;19:99–115. 22284358 10.1016/j.chembiol.2012.01.003 PMC3503453

[pro4970-bib-0032] Kudriaeva A , Kuzina ES , Zubenko O , Smirnov IV , Belogurov A . Charge‐mediated proteasome targeting. FASEB J. 2019;33:6852–6866. 30811957 10.1096/fj.201802237R

[pro4970-bib-0033] Kumar A , Maurya P , Hayes JJ . Post‐translation modifications and mutations of human linker histone subtypes: their manifestation in disease. IJMS. 2023;24:1463. 36674981 10.3390/ijms24021463 PMC9860689

[pro4970-bib-0034] Kwon M , Firestein BL . DNA transfection: calcium phosphate method. In: Zhou R , Mei L , editors. Neural development. Vol. 1018. Methods in molecular biology. Totowa, NJ: Humana Press; 2013. p. 107–110. 10.1007/978-1-62703-444-9_10 23681621

[pro4970-bib-0035] Leicher R , Osunsade A , Chua GNL , Faulkner SC , Latham AP , Watters JW , et al. Single‐stranded nucleic acid binding and coacervation by linker histone H1. Nat Struct Mol Biol. 2022;29:463–471. 35484234 10.1038/s41594-022-00760-4 PMC9117509

[pro4970-bib-0036] Li X , Amazit L , Long W , Lonard DM , Monaco JJ , O'Malley BW . Ubiquitin‐ and ATP‐independent proteolytic turnover of p21 by the REGγ‐proteasome pathway. Mol Cell. 2007;26:831–842. 17588518 10.1016/j.molcel.2007.05.028

[pro4970-bib-0037] Livak KJ , Schmittgen TD . Analysis of relative gene expression data using real‐time quantitative PCR and the 2−ΔΔCT method. Methods. 2001;25:402–408. 11846609 10.1006/meth.2001.1262

[pro4970-bib-0038] Lopez R , Sarg B , Lindner H , Bartolomé S , Ponte I , Suau P , et al. Linker histone partial phosphorylation: effects on secondary structure and chromatin condensation. Nucleic Acids Res. 2015;43:4463–4476. 25870416 10.1093/nar/gkv304 PMC4482070

[pro4970-bib-0039] Lu X , Hansen JC . Identification of specific functional subdomains within the linker histone H10 C‐terminal domain. J Biol Chem. 2004;279:8701–8707. 14668337 10.1074/jbc.M311348200

[pro4970-bib-0040] McConkey DJ . Calcium‐dependent, interleukin 1β‐converting enzyme inhibitor‐insensitive degradation of Lamin B1 and DNA fragmentation in isolated thymocyte nuclei. J Biol Chem. 1996;271:22398–22406. 8798402 10.1074/jbc.271.37.22398

[pro4970-bib-0041] Millán‐Ariño L , Izquierdo‐Bouldstridge A , Jordan A . Specificities and genomic distribution of somatic mammalian histone H1 subtypes. Biochim Biophys Acta. 2016;1859:510–519. 26477490 10.1016/j.bbagrm.2015.10.013

[pro4970-bib-0042] Murakami Y , Matsufuji S , Kameji T , Hayashi S , Igarashi K , Tamura T , et al. Ornithine decarboxylase is degraded by the 26S proteasome without ubiquitination. Nature. 1992;360:597–599. 1334232 10.1038/360597a0

[pro4970-bib-0043] Myers N , Olender T , Savidor A , Levin Y , Reuven N , Shaul Y . The disordered landscape of the 20S proteasome substrates reveals tight association with phase separated granules. Proteomics. 2018;18:1800076. 10.1002/pmic.201800076 30039638

[pro4970-bib-0044] Oh E , Mark KG , Mocciaro A , Watson ER , Prabu JR , Cha DD , et al. Gene expression and cell identity controlled by anaphase‐promoting complex. Nature. 2020;579:136–140. 32076268 10.1038/s41586-020-2034-1 PMC7402266

[pro4970-bib-0045] Pan C , Fan Y . Role of H1 linker histones in mammalian development and stem cell differentiation. Biochim Biophys Acta. 2016;1859:496–509. 26689747 10.1016/j.bbagrm.2015.12.002 PMC4775330

[pro4970-bib-0046] Peters JM , Franke WW , Kleinschmidt JA . Distinct 19 S and 20 S subcomplexes of the 26 S proteasome and their distribution in the nucleus and the cytoplasm. J Biol Chem. 1994;269:7709–7718. 8125997

[pro4970-bib-0047] Ponte I , Andrés M , Jordan A , Roque A . Towards understanding the regulation of histone H1 somatic subtypes with OMICs. J Mol Biol. 2021;433:166734. 33279581 10.1016/j.jmb.2020.166734

[pro4970-bib-0048] Ponte I , Vidal‐Taboada JM , Suau P . Evolution of the vertebrate H1 histone class: evidence for the functional differentiation of the subtypes. Mol Biol Evol. 1998;15:702–708. 9615451 10.1093/oxfordjournals.molbev.a025973

[pro4970-bib-0049] Qian M‐X , Pang Y , Liu CH , Haratake K , Du B‐Y , Ji D‐Y , et al. Acetylation‐mediated proteasomal degradation of Core histones during DNA repair and spermatogenesis. Cell. 2013;153:1012–1024. 23706739 10.1016/j.cell.2013.04.032 PMC3983474

[pro4970-bib-0050] Raghuram N , Strickfaden H , McDonald D , Williams K , Fang H , Mizzen C , et al. Pin1 promotes histone H1 dephosphorylation and stabilizes its binding to chromatin. J Cell Biol. 2013;203:57–71. 24100296 10.1083/jcb.201305159 PMC3798258

[pro4970-bib-0051] Ramakrishnan V , Finch JT , Graziano V , Lee PL , Sweet RM . Crystal structure of globular domain of histone H5 and its implications for nucleosome binding. Nature. 1993;362:219–223. 8384699 10.1038/362219a0

[pro4970-bib-0052] Roque A , Iloro I , Ponte I , Arrondo JLR , Suau P . DNA‐induced secondary structure of the carboxyl‐terminal domain of histone H1. J Biol Chem. 2005;280:32141–32147. 16006555 10.1074/jbc.M505636200

[pro4970-bib-0053] Roque A , Orrego M , Ponte I , Suau P . The preferential binding of histone H1 to DNA scaffold‐associated regions is determined by its C‐terminal domain. Nucleic Acids Res. 2004;32:6111–6119. 15562002 10.1093/nar/gkh945 PMC534626

[pro4970-bib-0054] Roque A , Ponte I , Arrondo JLR , Suau P . Phosphorylation of the carboxy‐terminal domain of histone H1: effects on secondary structure and DNA condensation. Nucleic Acids Res. 2008;36:4719–4726. 18632762 10.1093/nar/gkn440 PMC2504289

[pro4970-bib-0055] Sahu I , Mali SM , Sulkshane P , Xu C , Rozenberg A , Morag R , et al. The 20S as a stand‐alone proteasome in cells can degrade the ubiquitin tag. Nat Commun. 2021;12:6173. 34702852 10.1038/s41467-021-26427-0 PMC8548400

[pro4970-bib-0056] Sarg B , Lopez R , Lindner H , Ponte I , Suau P , Roque A . Identification of novel post‐translational modifications in linker histones from chicken erythrocytes. J Proteome. 2015;113:162–177. 10.1016/j.jprot.2014.10.004 25452131

[pro4970-bib-0057] Serna‐Pujol N , Salinas‐Pena M , Mugianesi F , Le Dily F , Marti‐Renom MA , Jordan A . Coordinated changes in gene expression, H1 variant distribution and genome 3D conformation in response to H1 depletion. Nucleic Acids Res. 2022;50:3892–3910. 35380694 10.1093/nar/gkac226 PMC9023279

[pro4970-bib-0058] Shabek N , Herman‐Bachinsky Y , Buchsbaum S , Lewinson O , Haj‐Yahya M , Hejjaoui M , et al. The size of the proteasomal substrate determines whether its degradation will Be mediated by mono‐ or polyubiquitylation. Mol Cell. 2012;48:87–97. 22902562 10.1016/j.molcel.2012.07.011

[pro4970-bib-0059] Shmueli MD , Sheban D , Eisenberg‐Lerner A , Merbl Y . Histone degradation by the proteasome regulates chromatin and cellular plasticity. FEBS J. 2022;289:3304–3316. 33914417 10.1111/febs.15903 PMC9292675

[pro4970-bib-0060] Singh RK , Liang D , Gajjalaiahvari UR , Kabbaj M‐HM , Paik J , Gunjan A . Excess histone levels mediate cytotoxicity via multiple mechanisms. Cell Cycle. 2010;9:4236–4244. 20948314 10.4161/cc.9.20.13636 PMC3055206

[pro4970-bib-0061] Talbert PB , Ahmad K , Almouzni G , Ausió J , Berger F , Bhalla PL , et al. A unified phylogeny‐based nomenclature for histone variants. Epigenetics Chromatin. 2012;5:7. 22650316 10.1186/1756-8935-5-7 PMC3380720

[pro4970-bib-0062] Ullrich O , Grune T . Proteasomal degradation of oxidatively damaged endogenous histones in K562 human leukemic cells. Free Radic Biol Med. 2001;31:887–893. 11585707 10.1016/s0891-5849(01)00672-4

[pro4970-bib-0063] Vila R , Ponte I , Collado M , Arrondo JLR , Jiménez MA , Rico M , et al. DNA‐induced α‐helical structure in the NH2‐terminal domain of histone H1. J Biol Chem. 2001;276:46429–46435. 11584004 10.1074/jbc.M106952200

[pro4970-bib-0064] Vila R , Ponte I , Jiménez MA , Rico M , Suau P . An inducible helix‐Gly‐Gly‐helix motif in the N‐terminal domain of histone H1e: a CD and NMR study. Protein Sci. 2002;11:214–220. 11790831 10.1110/ps.29602 PMC2373450

[pro4970-bib-0065] Voelkel‐Johnson C , Entingh AJ , Wold WS , Gooding LR , Laster SM . Activation of intracellular proteases is an early event in TNF‐induced apoptosis. J Immunol. 1995;154:1707–1716. 7836755

[pro4970-bib-0066] Xia Y , Yang W , Fa M , Li X , Wang Y , Jiang Y , et al. RNF8 mediates histone H3 ubiquitylation and promotes glycolysis and tumorigenesis. J Exp Med. 2017;214:1843–1855. 28507061 10.1084/jem.20170015 PMC5461008

[pro4970-bib-0067] Yasuda S , Tsuchiya H , Kaiho A , Guo Q , Ikeuchi K , Endo A , et al. Stress‐ and ubiquitylation‐dependent phase separation of the proteasome. Nature. 2020;578:296–300. 32025036 10.1038/s41586-020-1982-9

[pro4970-bib-0068] Zafar KS , Inayat‐Hussain SH , Ross D . A comparative study of proteasomal inhibition and apoptosis induced in N27 mesencephalic cells by dopamine and MG132. J Neurochem. 2007;102:913–921. 17504267 10.1111/j.1471-4159.2007.04637.x

[pro4970-bib-0069] Zhang Y , Cooke M , Panjwani S , Cao K , Krauth B , Ho P‐Y , et al. Histone H1 depletion impairs embryonic stem cell differentiation Copenhaver GP, editor. PLoS Genet. 2012;8:e1002691. 22589736 10.1371/journal.pgen.1002691 PMC3349736

[pro4970-bib-0070] Zlatanova JS , Srebreva LN , Banchev TB , Tasheva BT , Tsanev RG . Cytoplasmic pool of histone H1 in mammalian cells. J Cell Sci. 1990;96:461–468. 2229196 10.1242/jcs.96.3.461

