# Supplementary material for: Proteasome‐dependent degradation of histone H1 subtypes is mediated by its C‐terminal domain
Source: Protein Sci. 2024 Apr 9;33(5):e4970. doi: 10.1002/pro.4970 (PMC11002908; doi:10.1002/pro.4970)
Supplement: Supplementary file 1 — FIGURE S1. Inhibition of chymotrypsin activity after treatment with MG132 and BTZ. (a) Fluorescence spectra of Suc‐LLVY‐AMC peptide in solution (continuous line) and digested with chymotrypsin in vitro (dashed line). (b) Percentage of chymotrypsin activity in three biological replicates of T47D cells grown in presence of DMSO, 20 μM MG132 (MG132), and 20 nM Bortezomib (BTZ) for 12 h. The results are expressed as a percentage of the fluorescence emitted at 438 nm by Suc‐LLVY‐AMC peptide digested with chymotrypsin in vitro. Error bars correspond to the standard deviation. FIGURE S2. Accumulation of histone H1 subtypes upon proteasome inhibition in HeLa. (a) Western blot of H1 subtypes after treatment MG132, as described in materials and methods. (b) Quantification of the Western blot results in three biological replicates normalized by tubulin. Error bars correspond to the standard deviation. FIGURE S3. Inhibition of the proteasome and protein synthesis in T47D. Western blot of representative H1 subtypes after treatment with MG132 and cycloheximide, as described in materials and methods. Quantification of the western blot images in three biological replicates normalized by tubulin. Error bars correspond to the standard deviation. FIGURE S4. Protein stability of H1 subtypes in HeLa. (a) Western blot of H1 subtypes after treatment cycloheximide (CHX), as described in materials and methods. (b) Protein fraction in two biological replicates of each protein remaining after 8 h of treatment with CHX. Error bars correspond to the standard deviation. (c) Correlation between the accumulation after MG132 treatment and protein stability. r, correlation coefficient. FIGURE S5. Changes in the transcript levels of H1 subtypes after proteasome inhibition. Cells were treated with MG132, as described in materials and methods. RT‐qPCR results are expressed as fold change of the relative expression to GAPDH of each transcript in MG132 treated cells respect to the control cells grown in [file PRO-33-e4970-s001.pdf]

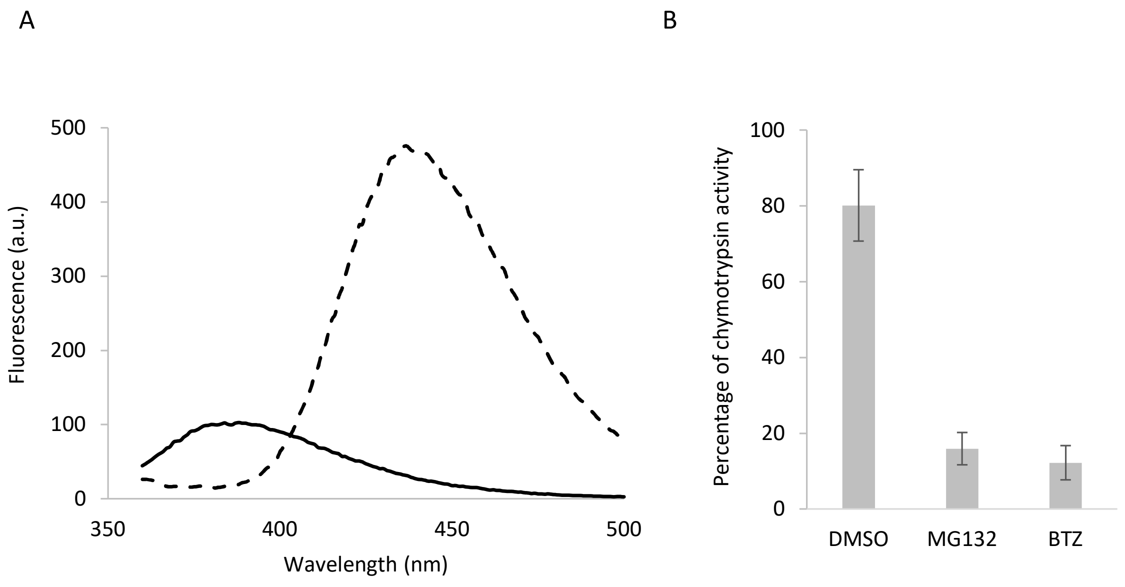

Figure S1. Inhibition of chymotrypsin activity after treatment with MG132 and BTZ. A, Fluorescence spectra of Suc-LLVY-AMC peptide in solution (continuous line) and digested with chymotrypsin in vitro (dashed line). B, Percentage of chymotrypsin activity in three biological replicates of T47D cells grown in presence of DMSO, 20 µM MG132 (MG132), and 20 nM Bortezomib (BTZ) for 12h. The results are expressed as a percentage of the fluorescence emitted at 438nm by Suc-LLVY-AMC peptide digested with chymotrypsin in vitro. Error bars correspond to the standard deviation.

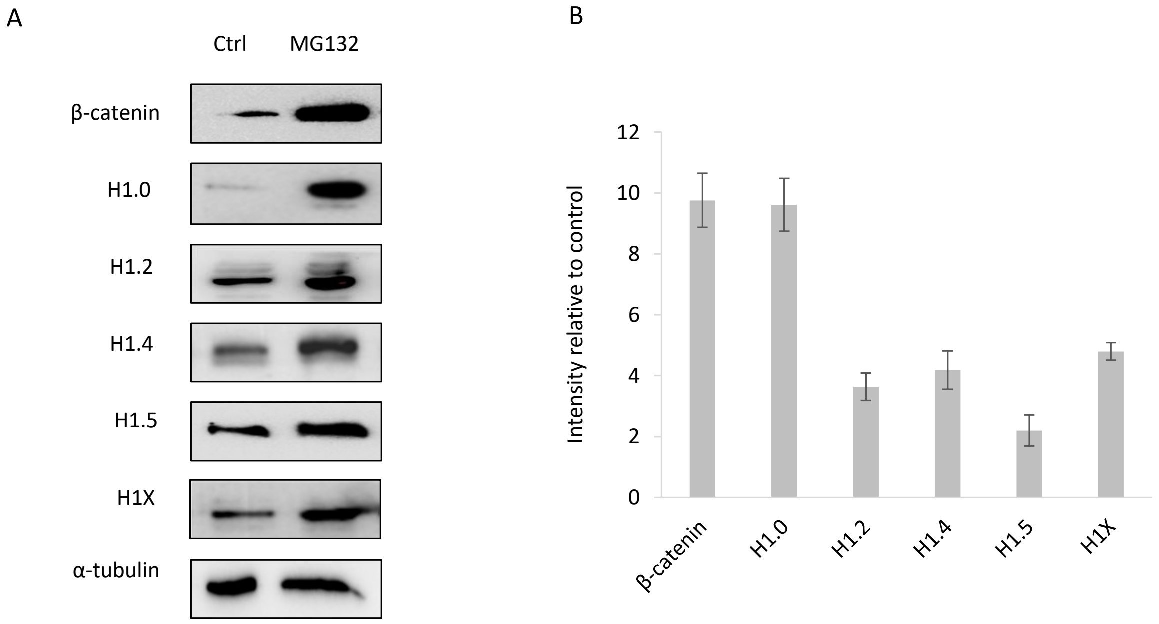

Figure S2. Accumulation of histone H1 subtypes upon proteasome inhibition in HeLa. A, Western blot of H1 subtypes after treatment MG132, as described in materials and methods. B, Quantification of the Western blot results in three biological replicates normalized by tubulin. Error bars correspond to the standard deviation.

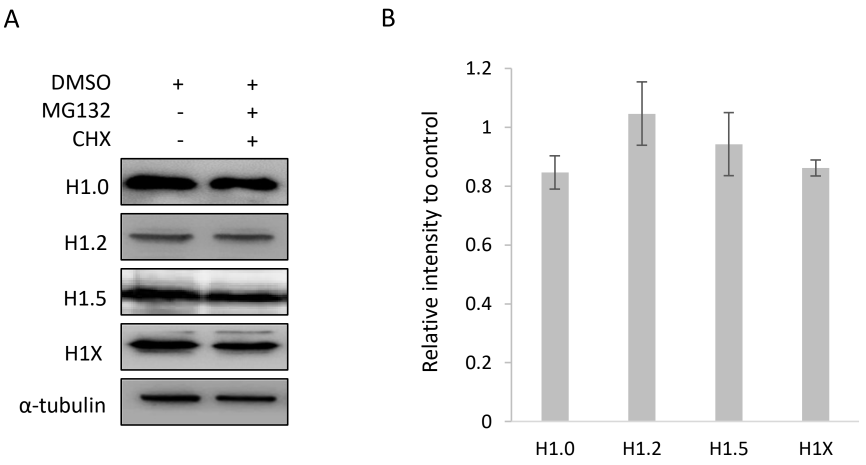

Figure S3: **Inhibition of the proteasome and protein synthesis in T47D.** Western blot of representative H1 subtypes after treatment with MG132 and cycloheximide, as described in materials and methods. Quantification of the western blot images in three biological replicates normalized by tubulin. Error bars correspond to the standard deviation.

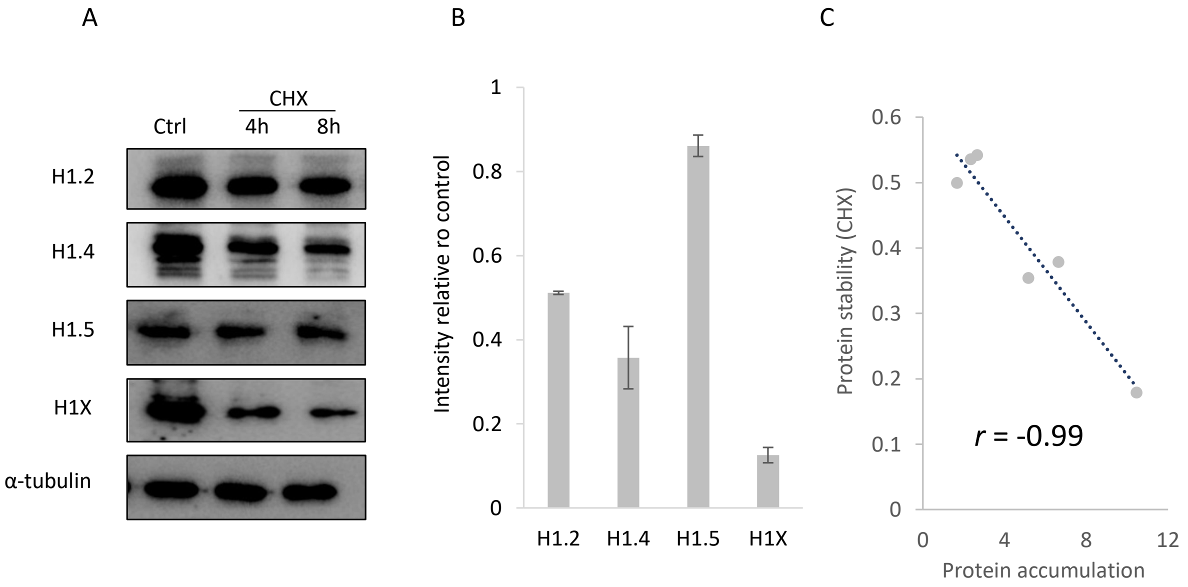

Figure S4. Protein stability of H1 subtypes in HeLa. A, Western blot of H1 subtypes after treatment cycloheximide (CHX), as described in materials and methods. B, Protein fraction in two biological replicates of each protein remaining after 8h of treatment with CHX. Error bars correspond to the standard deviation. C, correlation between the accumulation after MG132 treatment and protein stability. r, correlation coefficient.

A

B

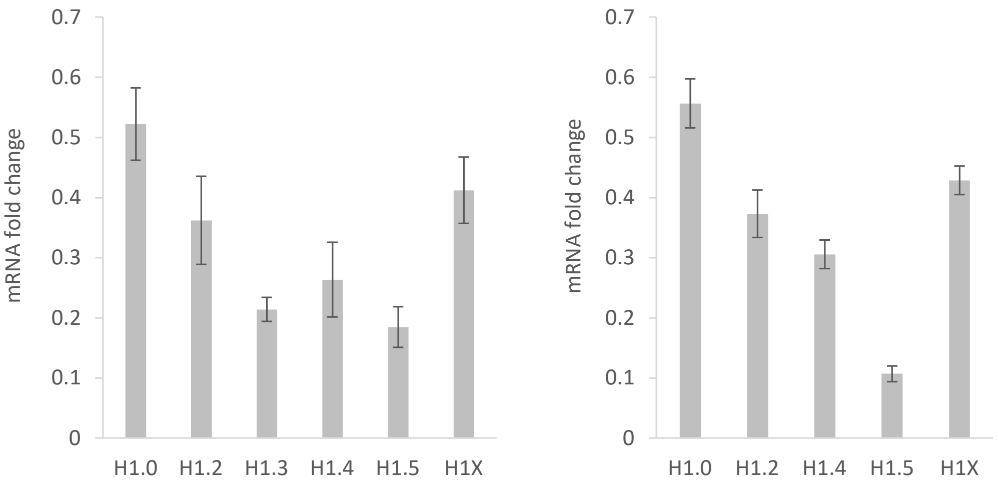

Figure S5. Changes in the transcript levels of H1 subtypes after protesome inhibition. Cells were treated with MG132, as described in materials and methods. RT-qPCR results are expressed as fold change of the relative expression to GAPDH of each transcript in MG132 treated cells respect to the control cells grown in media supplemented with 0.2 % DMSO of three biological replicates. A. T47D. B. HeLa. Error bars represent the standard deviation.

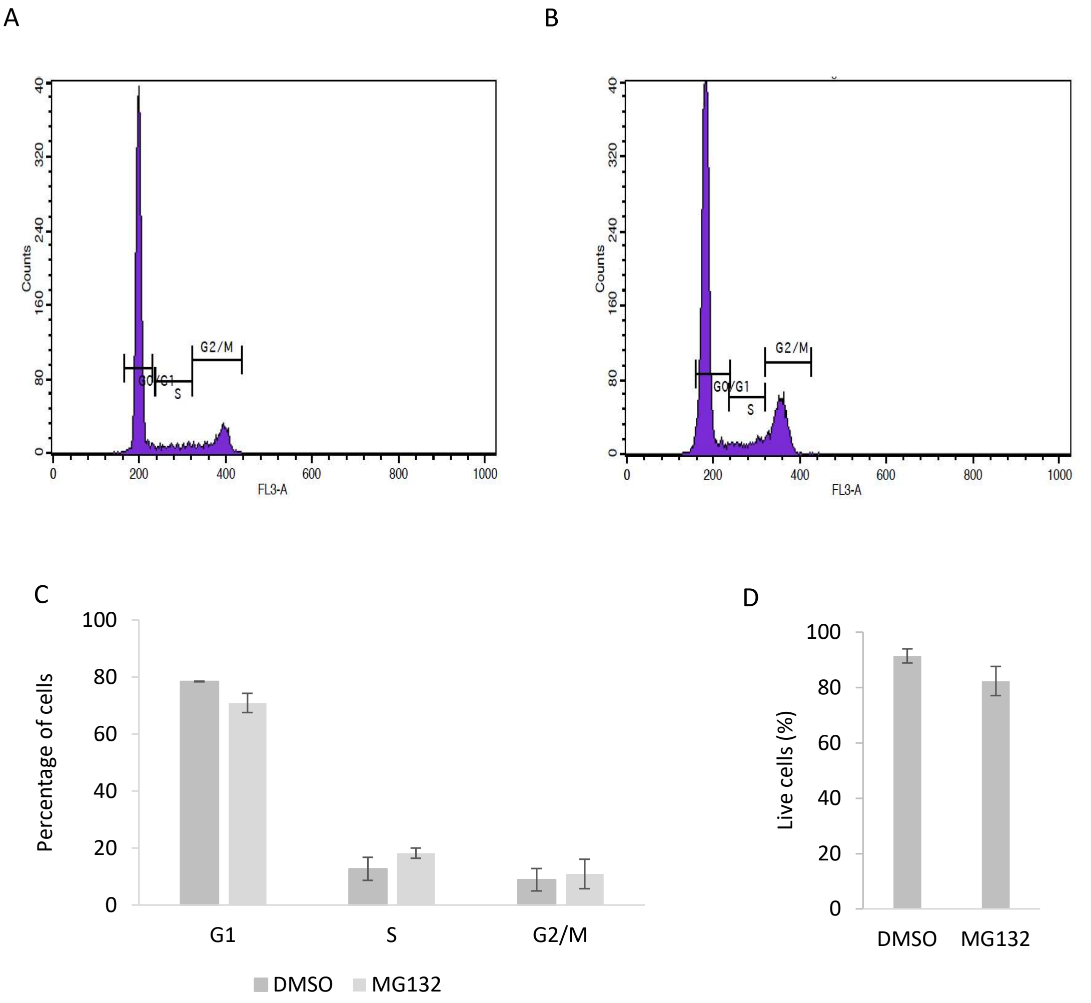

Figure S6. Effects of proteasome inhibition in T47D in cell cycle and survival. Flux cytometry profiling of cells stained with propidium iodine. A. control cells grown in 0.2% DMSO. B. cells treated with MG132, as described in material and methods. C. Proportion of cells in each phase in two biological replicates, expressed as percentages. D. Percentage of live cells after 12h in the presence of DMSO and MG132. Error bars correspond to the standard deviation.

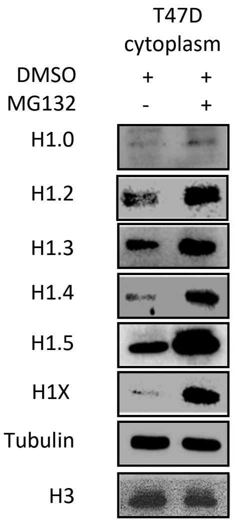

Figure S7. Accumulation of histone H1 subtypes in the cytoplasm after proteasome inhibition. Western Blots of a cytoplasmatic protein extract of T47D cells after treatment with MG132 (20µM 12h) .

A

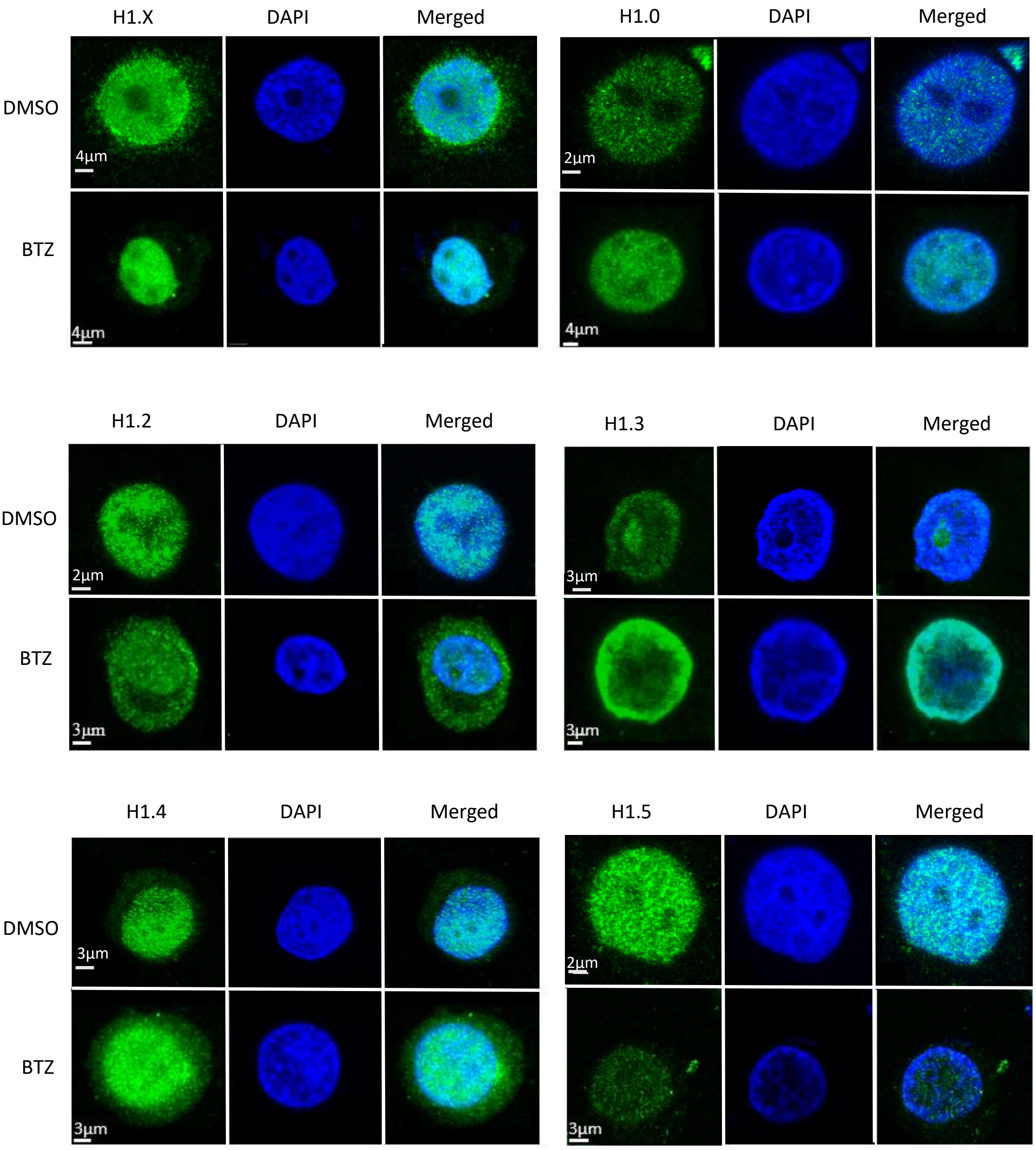

ns

B

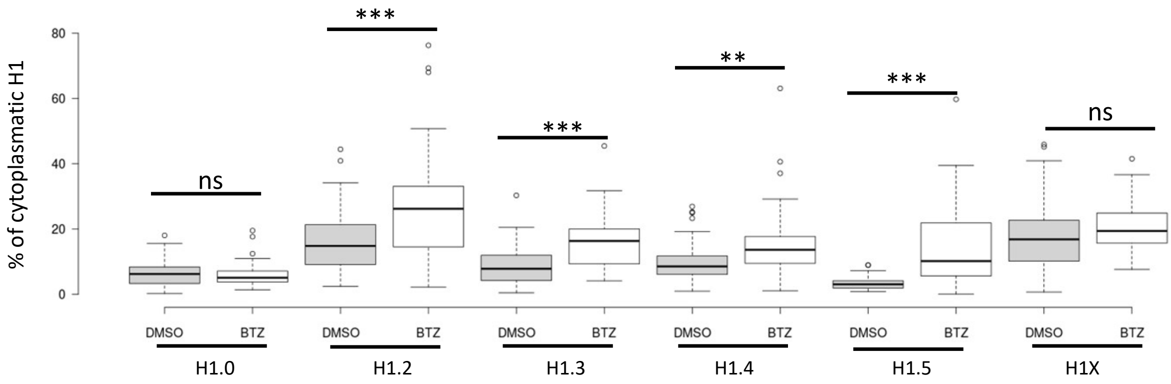

Figure S8. Accumulation of histone H1 subtypes in the cytoplasm of T47D cells after proteasome inhibition with Bortezomib. A. Representative immunofluorescence images of H1 somatic subtypes in T47D cells. Cells were treated with DMSO and Bortezomib (20nM in DMSO, 12h). Cellular nuclei were stained with DAPI. B. Box plots correspond to the quantification of 35-70 cells/variants and condition. Asterisks denote the p-value of the two-tailed Student's t-test showing the significance of the difference between untreated and treated cells * p-value < 0.05; ** p-value < 0.01; *** p-value < 0.001; n.s, not significant.

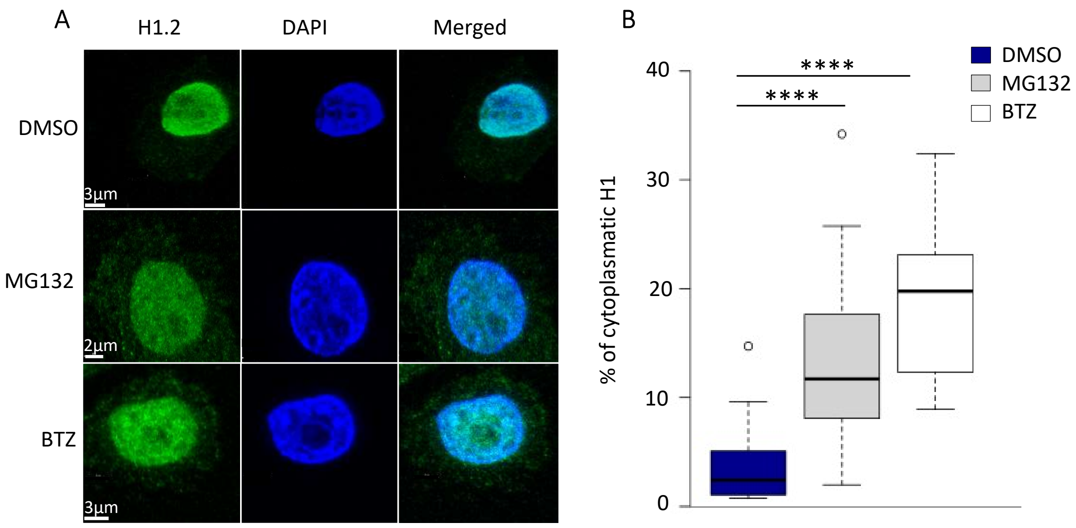

Figure S9. Accumulation of histone H1.2 in the cytoplasm of HeLa cells after proteasome inhibition. A. Representative immunofluorescence images of H1.2 in HeLa cells. Cells were treated with DMSO, MG132 (20 µM) and Bortezomib (20 nM) 12h. Cellular nuclei were stained with DAPI. B. Box plots correspond to the quantification of 35-70 cells/variants and condition. Asterisks denote the p-value of the two-tailed Student's t-test showing the significance of the difference between untreated and treated cells * p-value < 0.05; ** p-value < 0.01; *** p-value < 0.001; n.s, not significant.

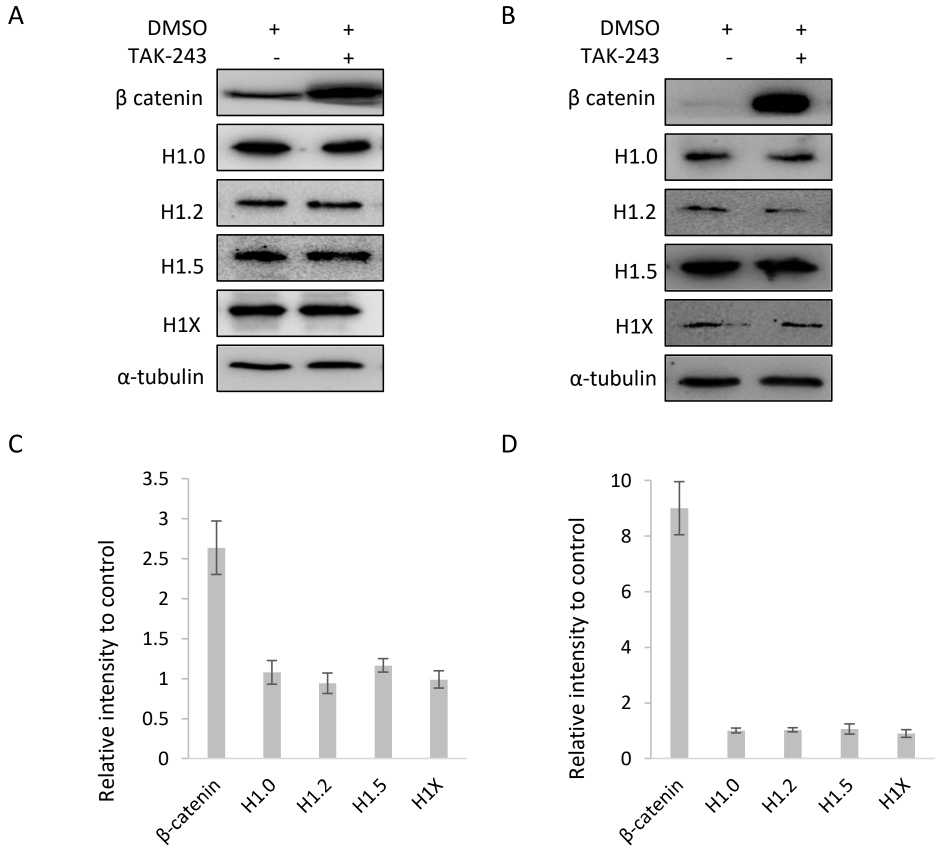

Figure S10. Inhibition of the ubiquitin pathway in human cell lines. Western blot images of T47D (A) and HeLa (B) cells treated with 5 µM TAK-243 for 12h. Quantification of the western blot images in three biological replicates normalized by tubulin in T47D (C) and HeLa (D). Error bars correspond to the standard deviation.

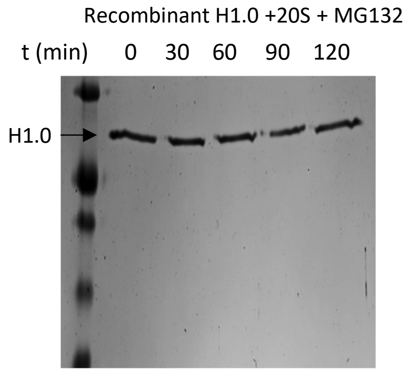

Figure S11. Inhibition of H1.0 degradation by the 20S proteasome in the presence of MG132. Silver staining of 15% SDS-PAGE of recombinant H1.0 incubated with the 20S proteasome and MG132 (20 µM).
